# TcMYC2 regulates Pyrethrin biosynthesis in *Tanacetum cinerariifolium*

**DOI:** 10.1093/hr/uhac178

**Published:** 2022-08-24

**Authors:** Tuo Zeng, Jia-Wen Li, Zhi-Zhuo Xu, Li Zhou, Jin-Jin Li, Qin Yu, Jin Luo, Zhu-Long Chan, Maarten A Jongsma, Hao Hu, Cai-Yun Wang

**Affiliations:** Key Laboratory for Biology of Horticultural Plants, Ministry of Education, College of Horticulture & Forestry Sciences, Huazhong Agricultural University, Wuhan 430070, China; School of Life Sciences, Guizhou Normal University, Guiyang 550000, China; Key Laboratory for Biology of Horticultural Plants, Ministry of Education, College of Horticulture & Forestry Sciences, Huazhong Agricultural University, Wuhan 430070, China; Key Laboratory for Biology of Horticultural Plants, Ministry of Education, College of Horticulture & Forestry Sciences, Huazhong Agricultural University, Wuhan 430070, China; Key Laboratory for Biology of Horticultural Plants, Ministry of Education, College of Horticulture & Forestry Sciences, Huazhong Agricultural University, Wuhan 430070, China; Key Laboratory for Biology of Horticultural Plants, Ministry of Education, College of Horticulture & Forestry Sciences, Huazhong Agricultural University, Wuhan 430070, China; Key Laboratory for Biology of Horticultural Plants, Ministry of Education, College of Horticulture & Forestry Sciences, Huazhong Agricultural University, Wuhan 430070, China; Key Laboratory for Biology of Horticultural Plants, Ministry of Education, College of Horticulture & Forestry Sciences, Huazhong Agricultural University, Wuhan 430070, China; Key Laboratory for Biology of Horticultural Plants, Ministry of Education, College of Horticulture & Forestry Sciences, Huazhong Agricultural University, Wuhan 430070, China; Business Unit Bioscience, Wageningen University and Research, Droevendaalsesteeg 1, 6708, PB Wageningen, the Netherlands; Key Laboratory for Biology of Horticultural Plants, Ministry of Education, College of Horticulture & Forestry Sciences, Huazhong Agricultural University, Wuhan 430070, China; Department of Botany and Plant Sciences, Institute of Integrative Genome Biology, University of California, Riverside, CA 92521, USA; Key Laboratory for Biology of Horticultural Plants, Ministry of Education, College of Horticulture & Forestry Sciences, Huazhong Agricultural University, Wuhan 430070, China

## Abstract

Pyrethrins constitute a class of terpene derivatives with high insecticidal activity and are mainly synthesized in the capitula of the horticulturally important plant, *Tanacetum cinerariifolium.* Treatment of *T. cinerariifolium* with methyl jasmonate (MeJA) in the field induces pyrethrin biosynthesis, but the mechanism linking MeJA with pyrethrin biosynthesis remains unclear. In this study, we explored the transcription factors involved in regulating MeJA-induced pyrethrin biosynthesis. A single spray application of MeJA to *T. cinerariifolium* leaves rapidly upregulated the expression of most known pyrethrin biosynthesis genes and subsequently increased the total pyrethrin content in the leaf. A continuous 2-week MeJA treatment resulted in enhanced pyrethrin content and increased trichome density. *TcMYC2*, a key gene in jasmonate signaling, was screened at the transcriptome after MeJA treatment. *TcMYC2* positively regulated expression of the pyrethrin biosynthesis genes *TcCHS*, *TcAOC*, and *TcGLIP* by directly binding to E-box/G-box motifs in the promoters. The stable overexpression of *TcMYC2* in *T. cinerariifolium* hairy roots significantly increased the expression of *TcAOC* and *TcGLIP*. Further transient overexpression and viral-induced gene-silencing experiments demonstrated that *TcMYC2* positively promoted pyrethrin biosynthesis. Collectively, the results reveal a novel molecular mechanism for MeJA-induced pyrethrin biosynthesis in *T. cinerariifolium* involving TcMYC2.

## Introduction

*Tanacetum cinerariifolium* is an economically important ornamental plant valued as a natural source of pyrethrins, a group of highly effective insecticidal secondary metabolites [1]. Natural pyrethrins have been widely used as an environmentally friendly insecticide, with low mammalian toxicity and no known insect immunity [2]. Consequently, *T. cinerariifolium* is cultivated in several countries, including Kenya, Tanzania, Uganda, Rwanda, China, and Australia [3], as a commercially important source of natural pesticides.

Natural pyrethrins comprise six types of monoterpene esters. The esterification involves an acid moiety (pyrethric acid and chrysanthemic acid) and an alcohol moiety (pyrethrolone, cinerolone, and jasomolone). The monoterpene acid moiety is derived from the methylerythritol-4-phosphate pathway and is exclusive to *T. cinerariifolium* and closely related species [4]. The first committed step is catalyzed by chrysanthemol synthase (CHS) [5, 6], which catalyzes the condensation of two dimethylallyl diphosphate (DMAPP) molecules to produce *trans*-chrysanthemol. Subsequently, *trans*-chrysanthemol is oxidized by alcohol dehydrogenase (ADH) and aldehyde dehydrogenase (ALDH) to produce *trans*-chrysanthemic acid [7]. Simultaneously, *trans*-chrysanthemal or *trans*-chrysanthemic acid is converted into pyrethric acid by chrysanthemol 10-hydroxylase (CHH) and 10-carboxychrysanthemic acid 10-methyltransferase [8]. Finally, GDSL lipase-like protein (GLIP) esterifies the acid and alcohol moieties into pyrethrins [9].

Synthesis of the alcohol moiety is at least partially shared with that of jasmonic acid (JA), a plant hormone that regulates the synthesis of plant secondary metabolites [10, 11]. Enzymes involved in steps of JA biosynthesis, including lipoxygenase (LOX), allene oxide synthase (AOS), allene oxide cyclase (AOC), and *oxo*-phytodienoic acid reductase, have been well established [10]. In the downstream pathway, jasmone hydroxylase (JMH) converts jasmone to jasmolone, and pyrethrolone synthase (PYS) catalyzes jasmolone to pyrethrolone [12, 13].

The pyrethrin content differs among organs in *T. cinerariifolium*. The pyrethrin content is ~1%–2% in the capitulum and 0.1% (dry weight) in leaves [14]. In industrial production, leaves are usually discarded and cause waste of feedstock. However, the content of pyrethrins in unexpanded leaves is relatively higher than in mature leaves and even comparable to that of capitula [15]. Mechanical damage may rapidly increase the pyrethrin content in leaves and lead to accumulation of JAs [16]. Therefore, there is potential to increase the pyrethrin content in leaves for commercial extraction and JAs might be crucial to promoting pyrethrinproduction. Methyl jasmonate (MeJA) regulates the biosynthesis of certain secondary metabolites in plants via transcription factors (TFs), such as phenolic acid and tanshinone in *Salvia miltiorrhiza* [17], artemisinin in *Artemisia annua* [18, 19], and flavor volatiles in *Chrysanthemum indicum* [20]. Typically, TFs positively or negatively regulate metabolite contents by directly binding to a downstream secondary metabolite biosynthesis target gene [21, 22]. MYC family proteins are master regulators of the JA signaling pathway [23]. Many MYC TFs respond to the MeJA signal and play critical roles in regulating terpenoid biosynthesis in diverse plant species [24, 25]. For instance, the JA-responsive TF *AsMYC2* positively regulates agarwood sesquiterpene synthesis by binding to the sesquiterpene synthase 1 (*ASS1*) promoter [26]. *AaMYC2* directly binds to the G-box motif in the promoters of *CYP71AV1* and *DBR2* to promote JA-mediated artemisinin biosynthesis [24]. In *Taxus chinensis*, *TcMYC2a* regulates taxol biosynthesis either directly or via ERF TFs depending on JA signaling [27].

In *T. cinerariifolium*, MeJA treatment and additional stimuli, such as mechanical wounding and volatile organic compounds, induce pyrethrin synthesis [16, 28, 29]. However, no TFs involved in the MeJA-mediated pyrethrin biosynthesis regulatory network have been reported in *T. cinerariifolium*. In this study, we found that short MeJA treatment applied to *T. cinerariifolium* leaves transiently induced the expression of pyrethrin biosynthetic genes and raised the pyrethrin content, and a continuous MeJA treatment even increased the trichome density, which further led to increasing pyrethrins content. Based on transcriptome analysis, we discovered a MeJA responsive *TcMYC2* TF which could directly bind to the promoter regions of key pyrethrins biosynthetic genes *TcCHS*, *TcAOC*, and *TcGLIP*. Further transient overexpression and virus-induced gene silencing (VIGS) of *TcMYC2* in *T. cinerariifolium* leaves confirmed that *TcMYC2* was a positive factor involved in regulating pyrethrin biosynthesis.

## Results

### Exogenous MeJA upregulates pyrethrin biosynthesis gene expression

**Figure 1:**
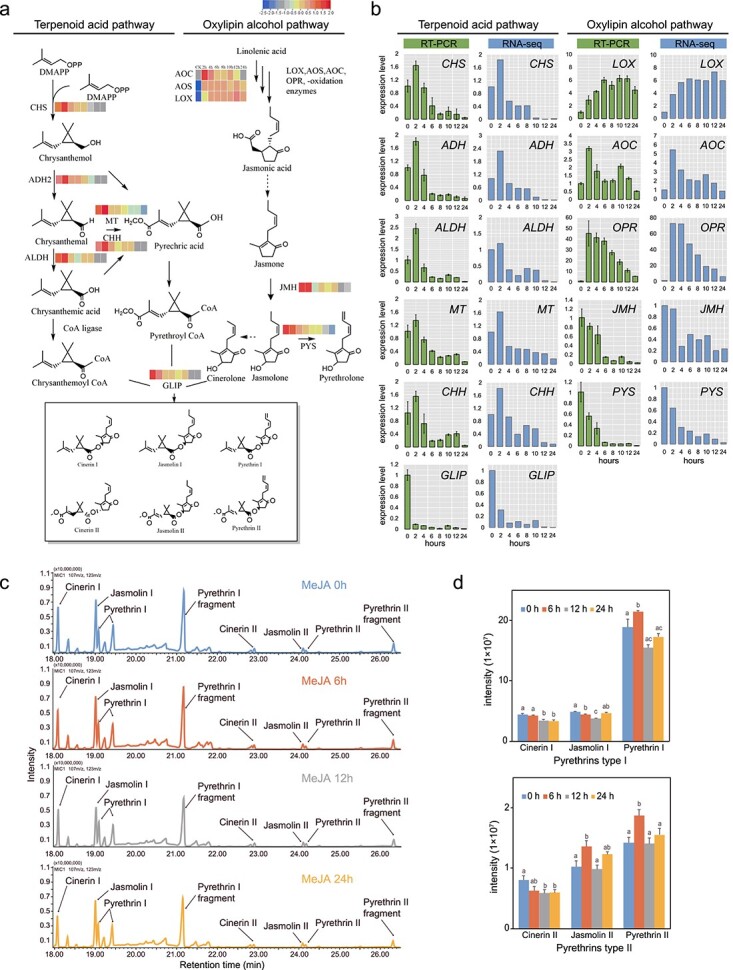
Pyrethrin biosynthesis gene expression and pyrethrin content in *Tanacetum cinerariifolium* leaves in response to short-term MeJA treatment. **a** Pyrethrin biosynthesis pathway. Heatmaps indicate the gene expression profile at different time points after MeJA treatment. Different colours represent the log_10_ FPKM value. Solid arrows indicate known steps in the pathway. Dotted arrows indicate steps not yet elucidated. The solid box encloses the six major pyrethrins. Abbreviations: ADH2, alcohol dehydrogenase2; ALDH, aldehyde dehydrogenase; CHH, chrysanthemol 10-hydroxylase; CHS, chrysanthemol synthase; GLIP, GDSL lipase-like protein; JMH, jasmolone hydroxylase; MT, 10-carboxychrysanthemic acid 10-methyltransferase; PYS, pyrethrolone synthase. **b** qRT-PCR analysis of pyrethrin biosynthesis genes. Blue bars are the fold change in average fragments per kilobase of transcript per million mapped reads (FPKM). Green bars are the qRT-PCR results normalized with the 2^−△△*C*t^ method using *GADPH* as an internal reference. The experiment was performed three times with three biological replicates. Error bars represent mean ± SD. **c** GC–MS chromatograms of leaves treated for 0, 6, 12, and 24 h. The relative pyrethrin content was determined using tetradecane as an internal standard, with three biological replicates. **d** Relative contents of total pyrethrins. The *y*-axis represents peak area intensity. Error bars represent the SD of the mean of three replicates. Different lower-case letters indicate a significant difference (*P* < 0.05) from one-way ANOVA followed by a *post-hoc* Tukey test.

Pyrethrins are esters of an acid moiety and an alcohol moiety derived from two pathways: an irregular monoterpene biosynthesis pathway (specific to *T. cinerariifolium*) and the JA biosynthesis pathway (Fig. 1a). To investigate the mechanism of MeJA-induced pyrethrin biosynthesis, the transcriptome of *T. cinerariifolium* leaves was analysed at 0 (control), 2, 4, 6, 8, 10, 12, and 24 h after a single spray application of MeJA, and differentially expressed genes (DEGs) associated with pyrethrin synthesis were identified. Transcriptome assembly and analysis details are presented in Figs S1 and S2, see online supplementary material. Expression levels of *TcLOX* (KC441523.1), *TcAOS* (MH397471.1), and *TcAOC* (MH397472.1), which function upstream of JA, rapidly increased at 2 h and were maintained at a higher level compared with the control (Fig. 1b). The monoterpene pathway genes *TcCHS* (JX913536.1), *TcADH* (MF497444.1), *TcALDH* (MF497445.1), *TcCHH* (KC441525.1), and *TcMT* (MK139710.1) were strongly upregulated within 2 h but thereafter were dramatically downregulated to levels much lower than those of the control (Fig. 1b). We speculated that although the short MeJA treatment could transiently and sharply induce the pyrethrins biosynthesis genes, the expression of these genes, especially which involved the synthesis of the acid moiety unique to *T. cinerariifolium*, was finally downregulated. Moreover, the expression of genes involved in the final steps of pyrethrin biosynthesis, including *TcJMH* (MG189934.1), *TcPYS* (MG874680.1), and *TcGLIP* (JN418994.1), was decreased in response to MeJA treatment, and we speculate this may be due to feedback regulation to maintain cell homeostasis (Fig. 1b). The expression patterns of these genes was confirmed by real-time quantitative reverse-transcription PCR (qRT-PCR) (Fig. 1b).

### Exogenous MeJA increases pyrethrin content in leaves

Methyl jasmonate stimulates overaccumulation of many secondary metabolites in plants [11]. Therefore, gas chromatography–mass spectrometry (GC–MS) analysis was conducted to detect pyrethrin accumulation in leaves of MeJA-treated plants (Fig. 1c). Contents of pyrethrin compounds, especially pyrethrin I and pyrethrin II, were significantly increased at 6 h after MeJA treatment, consistent with the timing of pyrethrin biosynthesis gene expression. At subsequent time points, the pyrethrin contents declined to levels lower than those at 0 h consistent with the gene expression profiles (Fig. 1d). Hence, we concluded that MeJA could promote pyrethrins production in response to a transient upregulation of pyrethrins biosynthetic genes; however, it did not maintain the higher pyrethrins content over time by a transient MeJA induction.

### *TcMYC2* is a potential regulator of MeJA-induced pyrethrin biosynthesis

To examine the role of JA pathway genes in pyrethrin biosynthesis, based on the FPKM expression matrix of 28 552 DEGs, a weighted gene coexpression network analysis (WGCNA) module was constructed. The 33 modules were obtained with a shear value of 0.2 (Fig. S3). The Cyan module (2320 genes) and Skyblue1 (211 genes) module were strongly correlated with pyrethrin synthesis. KEGG and GO enrichment analysis confirmed the genes were involved in secondary metabolite biosynthesis (Fig. S4). To explore the key regulatory genes, a candidate hub gene network was visualized with Cytoscape. Among the hub genes showing high connectivity, *TcMYC2* was detected (Fig. S4).

**Figure 2:**
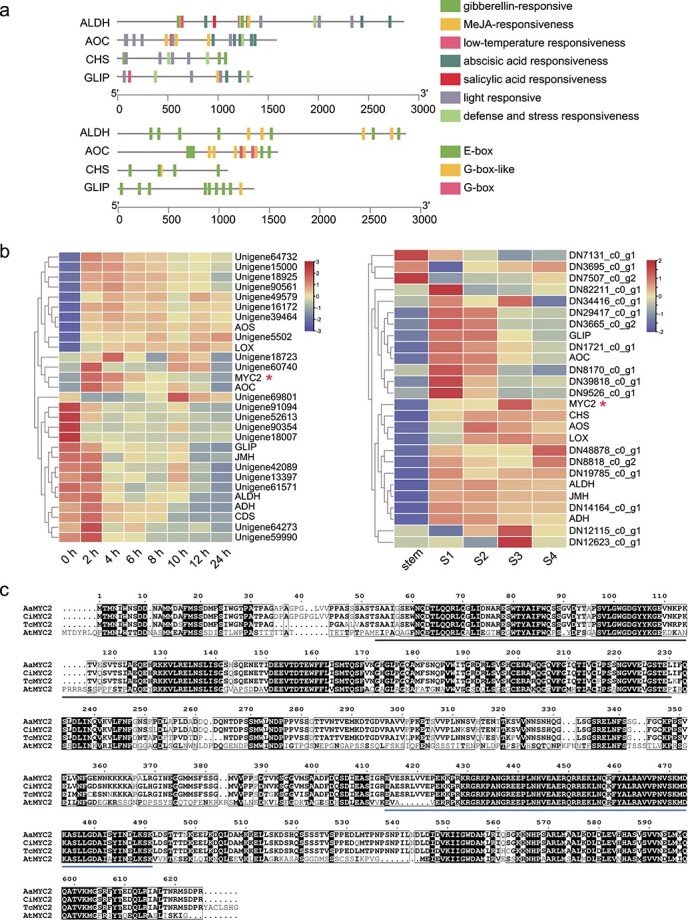
Bioinformatic analysis of pyrethrin biosynthesis gene promoters and expression profile of bHLH and pyrethrin biosynthesis genes. **a***Cis*-regulatory elements in promoters of key pyrethrin biosynthesis genes. **b** Heat maps of coexpressed genes based on RNA-Seq analyses (at time points after MeJA treatment of the leaves and at four flower developmental stages). **c** Alignment of amino acid sequences for TcMYC2 and homologous proteins. *Artemisia annua MYC2* (AKO62850.1); *Chrysanthemum indicum MYC2* (QED22045.1); *Arabidopsis thaliana MYC2* (NP_174541.1). The conserved MYC domains are indicated by lines below sequences. Black line, bHLH_MYC_N domain; blue line, HLH domain.

To identify the TFs activated by MeJA that potentially regulated pyrethrin biosynthesis, the promoter sequences of *TcCHS*, *TcALDH*, *TcAOC*, and *TcGLIP* were cloned and putative TF-binding sites in the promoter sequences was analysed using PlantCARE and PlantPAN3.0 (Fig. 2a). MeJA-responsive elements were detected in the *TcALDH*, *TcAOC*, and *TcGLIP* promoters. In addition, E-box and G-box motifs, the binding sites of bHLH TFs, were identified in the promoter of each gene. Fifty-five bHLH TFs with FPKM >1 were identified with PlantTFDB (Fig. S3). To obtain candidate MeJA-regulated bHLHs regulating pyrethrin biosynthesis, a heatmap was constructed using the RNA-seq data for pyrethrin biosynthesis genes obtained from the MeJA treatment and different development stages (Fig. 2b). Based on Pearson distance measurement and Ward clustering algorithm, the candidate unigene66762 showed the most similar expression pattern to *TcCHS* or *TcAOC*. Phylogenetic analysis of bHLHs showed that unigene66762 belonged to the MYC2-like subclade (Figs S5 and S6, see online supplementary material) and contained a conserved MYC domain (Fig. 2c). Thus, we named unigene66762 as *TcMYC2* and selected it for further analysis*.*

### Expression profiles and subcellular localization of *TcMYC2*

**Figure 3:**
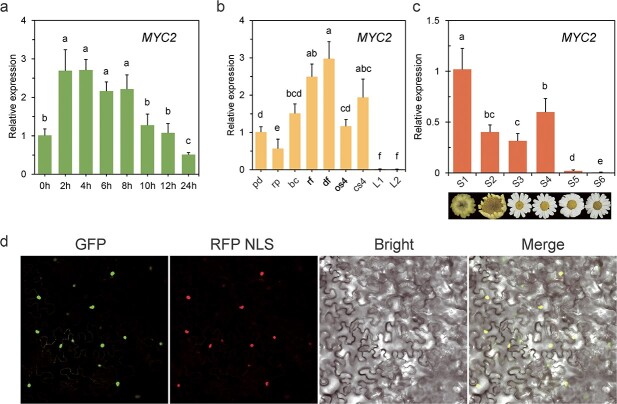
Expression profile and subcellular localization of *TcMYC2*. **a** qRT-PCR analysis of transcript abundance of *TcMYC2* induced in leaves of MeJA-treated 1-month-old tissue-cultured seedlings with three biological replicates. The fold change in relative expression level was normalized to the 0 h nontreated samples. Error bars represent mean ± SD with three biological replicates and two technical replicates. Different lower-case letters indicate a significant difference (*P* < 0.05) from one-way ANOVA followed by a *post-hoc* Tukey test. **b** qRT-PCR analysis of *TcMYC2* transcript abundance in leaf and flower tissues at different developmental stages, normalized against the pd transcript level. S2 flower stages (pd: pedicel of immature bud; rp: receptacle; bc: involucral bract; rf: ray floret; df: disc floret); S4 flower stages (os4: fully open ray floret, cs4: unopened ray floret); L1: immature developing leaf; L2: mature leaf. Different letters indicate a significant difference (*P* < 0.05) from one-way ANOVA followed by a *post-hoc* Tukey test. **c** qRT-PCR analysis of *TcMYC2* transcript abundance from S1 to S6 stages, normalized against the S1 transcript level. Different letters indicate a significant difference (*P* < 0.05) from one-way ANOVA followed by a *post-hoc* Tukey test. **d** Subcellular localization of *TcMYC2* in *N. benthamiana* leaf epidermal cells. Bright: brightfield image; GFP: GFP fluorescence of TcMYC2-GFP fusion protein; Merge: merged image; RFP NLS: RFP fluorescence of nuclear marker fusion protein.

To validate MeJA-mediated induction of *TcMYC2* expression, qRT-PCR was used to analyse *TcMYC2* expression. The *TcMYC2* transcript abundance strongly increased at 2 h, and was maintained at a high level after prolonged MeJA exposure (4, 6, and 8 h), until returning to the original level at 24 h (Fig. 3a). These results were consistent with the expression pattern of *TcAOC* in MeJA-treated leaves (Fig. 1b). To investigate potential similarities in expression patterns between *TcMYC2* and other pyrethrin biosynthesis genes, the relative expression levels of *TcMYC2* were analysed in different tissues and flowers at different developmental stages. The *TcMYC2* expression level was lowest in leaves, low in the receptacle and pedicel, and highest in ray florets and disc florets (Fig. 3b). The expression profiles of *TcMYC2* at different stages of flowering (Fig. 3c) were similar to those of other pyrethrin biosynthesis genes, including *TcCHS*, *TcALDH*, and *TcGLIP*, which are highly expressed at the first two stages of flowering. These results suggest that *TcMYC2* is involved in the regulation of pyrethrins biosynthesis. Thus, we speculated that *TcMYC2* co-regulates pyrethrin production by interacting with pyrethrins biosynthetic gene promoters.

To determine the subcellular localization of *TcMYC2*, the coding sequence of TcMYC2 was fused in-frame to the green fluorescent protein (GFP) under the control of the *Cauliflower mosaic virus* (CaMV) *35S* promoter. When *TcMYC2-GFP* was transiently expressed in *Nicotiana benthamiana* leaves, strong fluorescence was localized to the nucleus. The GFP signal co-localized with the RFP nuclear localization signal (NLS; Fig. 3d). These results indicate that TcMYC2 is a nuclear-localized protein according to its potential role as a TF.

### *TcMYC2* activates and directly binds to *TcCHS*, *TcAOC*, and *TcGLIP* promoters

**Figure 4:**
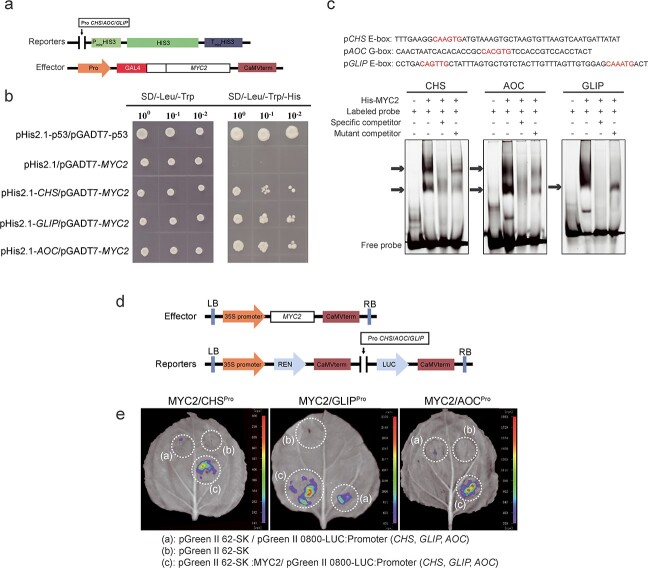
TcMYC2 directly binds to and activates the promoters of *TcCHS*, *TcAOC*, and *TcGLIP*. **a** Schematic diagram of Y1H vectors. Reporters: the promoter of *TcCHS*/*TcAOC*/*TcGLIP* was inserted into the pHis2.1 vector. Effector: *TcMYC2* was inserted into the pGADT7 vector. **b** Y1H assay showing TcMYC2 binding to *TcCHS*/*TcAOC*/*TcGLIP* promoter fragments. Growth on SD/−Leu/−Trp and SD/−Leu/−Trp/−His medium in a 10-fold dilution series. pHis2.1/pGADT7-*TcMYC2* was used as a negative control, pHis2.1-*p53*/pGADT7-*p53* was used as a positive control. **c** EMSA of interaction between TcMYC2 and the promoters of *TcCHS*/*TcAOC*/*TcGLIP*. The probe was designed from a fragment of the promoter containing E-box or G-box motifs. Purified His-tagged TcMYC2 protein solution was incubated with 10 μM FAM-labeled probe. Non-labeled specific cold probes and mutated cold probes at 50-fold dilution were used for the competition test. The shifted bands are indicated with an arrow. **d** Schematic diagram of dual-luciferase (LUC) vectors. Effector: *TcMYC2* was inserted into the pGreen-62-SK vector driven by the CAMV *35S* promoter. Reporters: the promoters of *TcCHS*/*TcAOC*/*TcGLIP* were inserted into the pGreen II-0800-LUC vector and drove LUC RNA translation. **e** Transient expression assays showing TcMYC2 binding to the *TcCHS*/*TcAOC*/*TcGLIP* promoters and promotion of Luc expression. (a), pGreen-62-SK/pGreen II-0800-LUC-*TcCHS*/*TcAOC*/*TcGLIP* promoters; (b), pGreen-62-SK empty vector as a negative control; (c), pGreen-62-SK-MYC2/pGreen II-0800-LUC-*TcCHS*/*TcAOC*/*TcGLIP* promoters.

Given that binding sites of bHLH TFs were predicted in the *TcCHS*, *TcALDH*, *TcAOC*, and *TcGLIP* promoter sequences, and the *TcMYC2* expression pattern was similar to that of four pyrethrin biosynthesis genes, yeast one-hybrid (Y1H) assays were conducted to test the hypothesis that *TcMYC2* regulates the expression of these genes by directly binding to the corresponding promoter. The promoter of each gene was cloned and inserted into the pHis2.1 vector to generate reporter constructs. *TcMYC2* was fused to the GAL4 activation domain to generate the effector construct pGADT7-*TcMYC2* (Fig. 4a). The reporter and effector constructs were co-transformed into yeast cells. Yeast grew on SD/−Leu/−Trp/−His medium only when pGADT7-*TcMYC2* was co-transformed with pHis2.1-*TcCHS*, pHis2.1-*TcAOC*, or pHis2.1-*TcGLIP* but not with pHis2.1, suggesting that TcMYC2 can directly bind to the *TcCHS*, *TcAOC*, and *TcGLIP* promoters (Fig. 4b). The direct binding motif in the promoters was verified by electrophoretic mobility shift assays (EMSAs). The shifted band was separately detected in the presence of both TcMYC2 and a labeled probe containing the G-box in the *TcAOC* promoter or E-box in the *TcCHS* and *TcGLIP* promoters (Fig. 4c). The intensity of the shifted band decreased with addition of 50-fold concentrations of cold competitor (unlabeled probe) and recovered with addition of mutant competitor probe.

To verify the interaction of TcMYC2 with the *TcCHS*, *TcAOC*, and *TcGLIP* promoters *in vivo*, transient dual-luciferase (dual-LUC) assays were performed. Reporter constructs (*TcCHS*/*TcAOC*/*TcGLIP*pro:*LUC*) and effector constructs (*35S*:*TcMYC2*) were transiently coexpressed in *N. benthamiana* leaves (Fig. 4d). Strong LUC activity was observed in leaves co-transformed with *TcCHS*/*TcAOC*/*TcGLIP*pro:*LUC* and *35S*:*TcMYC2*. No signal was detected in leaves transformed with *TcCHS*/*TcAOC*/*TcGLIP*pro:*LUC* or the empty vector (Fig. 4e). Collectively, we demonstrated that TcMYC2 directly activates these gene promoters and regulates pyrethrins biosynthesis in *T. cinerariifolium*.

### Stable transformation of *TcMYC2* in hairy roots

**Figure 5:**
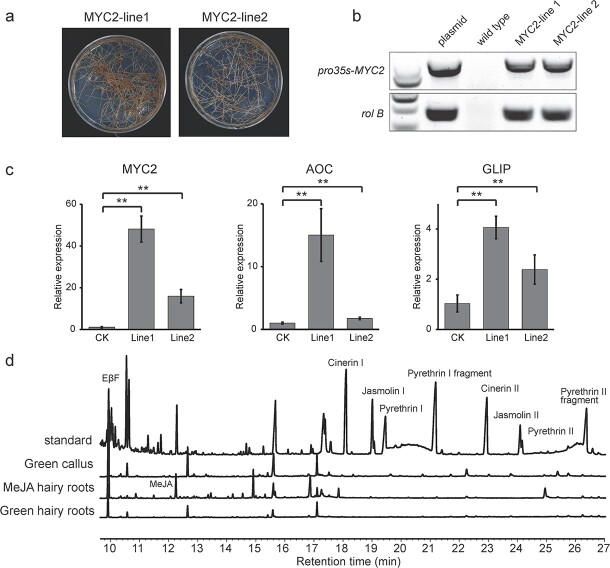
Analysis of overexpression of *TcMYC2* in transgenic hairy roots. **a** Resistant hairy roots of two lines were cultured onto selective medium containing kanamycin (10 mg/L) to propagate for approximately 30 d. **b** Detection of *rolB* and *35 s-TcMYC2* by PCR. **c** Gene expression in transgenic hairy roots. Values are the mean ± SD. ^*^P < 0.05, ^**^P < 0.01 (two-tailed Student’s *t-*test). **d** GC–MS analysis of transgenic hairy roots. Standard: commercial pyrethrum extract standard; Green callus: TcMYC2-transgenic green callus cultured under a normal photoperiod (16 h day, 8 h night) for 30 d; MeJA hairy roots: *TcMYC2*-transgenic calli treated with MeJA for 3 d; Green hairy roots: *TcMYC2*-transgenic green hairy roots cultured under a normal photoperiod (16 h day, 8 h night) for 30 d.

To verify the function of stably overexpressed *TcMYC2*, *TcMYC2* was inserted in the pBI121 vector, and the recombinant vector was transferred to *T. cinerariifolium* via *Agrobacterium rhizogenes* to obtain stable transgenic hairy roots. After 30 d (Fig. 5a), positive transgenic hairy roots were selected via PCR amplification of *rolB* and *35 s-TcMYC2* (Fig. 5b). We first checked the gene expression of *TcMYC2* and its target genes TcCHS, TcAOC, and TcGLIP in transgenic hairy root. As expected, *TcAOC* and *TcGLIP* expression was significantly upregulated, suggesting that *TcMYC2* may positively regulate its target genes (Fig. 5c). However, the *C*_t_ value of *TcCHS* was close to 40 and was barely expressed in hairy roots, owing to the lack of trichome tissue in which *TcCHS* is specifically expressed [30]. The GC–MS analysis revealed that no pyrethrins were produced in hairy roots except for (*E*)-β-farnesene (EβF), which shares the substrate DMAPP with pyrethrins. Thus, *TcCHS* expression in the trichome was indispensable for *de novo* pyrethrin biosynthesis even though the substrate was supplied. No pyrethrins were detected in stable transformed green callus, which further supported this hypothesis (Fig. 5d). Thus, based on stable transformation in hairy roots, *TcMYC2* positively upregulated its target genes *TcAOC* and *TcGLIP* in *T. cinerariifolium.*

### Transient overexpression of *TcMYC2* upregulates pyrethrin biosynthesis genes and pyrethrin accumulation

**Figure 6:**
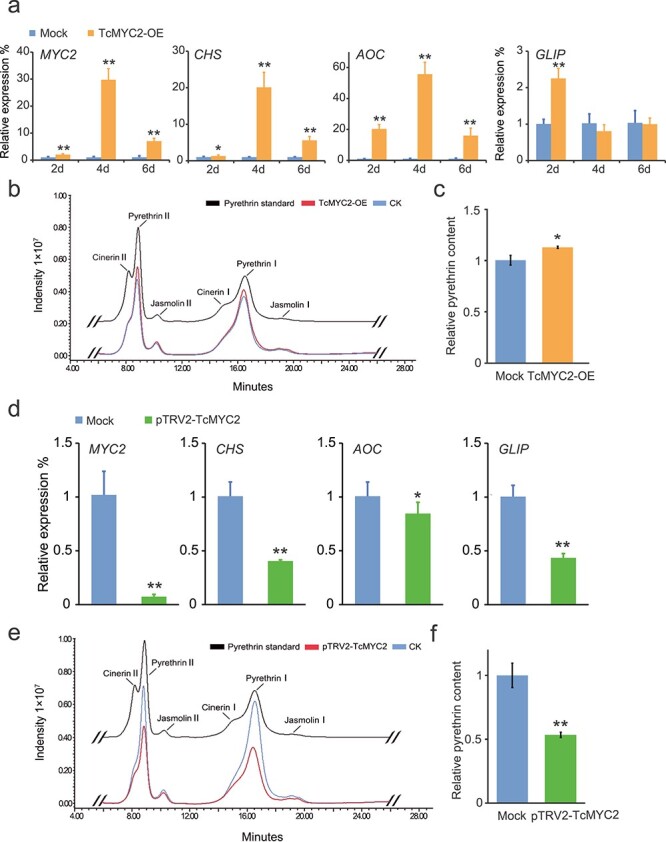
Transient overexpression and TRV-VIGS treatment of TcMYC2 in *Tanacetum cinerariifolium* leaves. **a** Pyrethrin biosynthesis gene expression in *T. cinerariifolium* leaves at 2, 4, and 6 d after transient overexpression of *TcMYC2*. Mock: transient overexpression of the empty vector; TcMYC2-OE: transient overexpression of pGreen-62-SK-TcMYC2. The fold change in relative expression level was normalized to the mock expression. **b** Representative HPLC chromatogram of total pyrethrins extracted from leaves after transient *TcMYC2* overexpression. CK: transient overexpression of the empty vector; TcMYC2-OE: transient overexpression of pGreen-62-SK-TcMYC2. **c** Pyrethrin content in *T. cinerariifolium* leaves after transient *TcMYC2* overexpression at 4 d. The ordinate represents the intensity peak area detected by HPLC; the abscissa represents the six major pyrethrin compounds. **d** Pyrethrin biosynthesis gene expression in *T. cinerariifolium* leaves after TRV-VIGS treatment of *TcMYC2* at 14 d. Mock: VIGS empty pTRV2 vector; pTRV2-TcMYC2: TcMYC2 silenced leaves. The fold change in relative expression level was normalized to the mock expression. **e** Representative HPLC chromatogram of total pyrethrins extracted from TRV-VIGS treatment leaves. CK: VIGS empty pTRV2 vector; pTRV2-TcMYC2: TcMYC2 silenced leaves. **f** Pyrethrin content in *T. cinerariifolium* leaves after TRV-VIGS treatment of *TcMYC2*. The ordinate represents the intensity peak area detected by HPLC; the abscissa represents the six major pyrethrin compounds. Values are the mean ± SD of three biological replicates. ^*^P < 0.05, ^**^P < 0.01 (two-tailed Student’s *t-*test).

The role of TcMYC2 in regulating pyrethrin biosynthesis in *T. cinerariifolium* was investigated. Given that stable transformation of *T. cinerariifolium* is not available and to mimic short-term MeJA-induced upregulation of pyrethrin biosynthesis, we transiently overexpressed *TcMYC2* in *T. cinerariifolium* leaves, and determined the change in expression of pyrethrin biosynthesis genes by qRT-PCR at 2, 4, and 6 d post transformation. Compared with mock plants transformed with blank pGreenII62-SK, *TcMYC2* transcript abundance was significantly increased in transformed plants, peaking at 4 d, and thereafter declined; however, at 6 d post transformation, *TcMYC2* transcript abundance in transformed plants remained higher than that in the mock plants (Fig. 6a). Expression of *TcCHS* and *TcAOC* was markedly elevated in *TcMYC2*-overexpressing plants, peaking at 4 d, which was 11.7- to 55.7-fold higher than that in the mock plants (Fig. 6a). Expression of *TcGLIP* increased by 2.2-fold at 2 d compared with the control, and thereafter decreased to levels lower than the control (Fig. 6a). Significant differences were detected in the total pyrethrin contents between *TcMYC2*-overexpressing and mock leaves (Fig. 6bc). These results demonstrated that overexpression of *TcMYC2* enhanced pyrethrin accumulation.

### Transient silencing of *TcMYC2* decreases pyrethrin biosynthesis gene expression and pyrethrin accumulation

A viral-induced gene silencing (VIGS) assay was conducted to confirm the indispensable role of *TcMYC2* in regulating pyrethrin biosynthesis. Compared with the mock (empty pTRV2 vector), *TcMYC2* expression in *T. cinerariifolium* leaves was significantly reduced by ~93% in silenced plants (Fig. 6d). Downregulation of *TcMYC2* expression resulted in a significant decrease (~1.2- to 2.5-fold) in the expression level of *TcCHS*, *TcAOC*, and *TcGLIP* (Fig. 6d). The total pyrethrin content was also reduced in *TcMYC2*-silenced *T. cinerariifolium* leaves (Fig. 6). These results suggest that *TcMYC2* is an important positive regulator of pyrethrins biosynthesis, which acts by directly activating the pyrethrins biosynthetic genes.

### Continuous MeJA application increases trichome density and pyrethrin content

**Figure 7:**
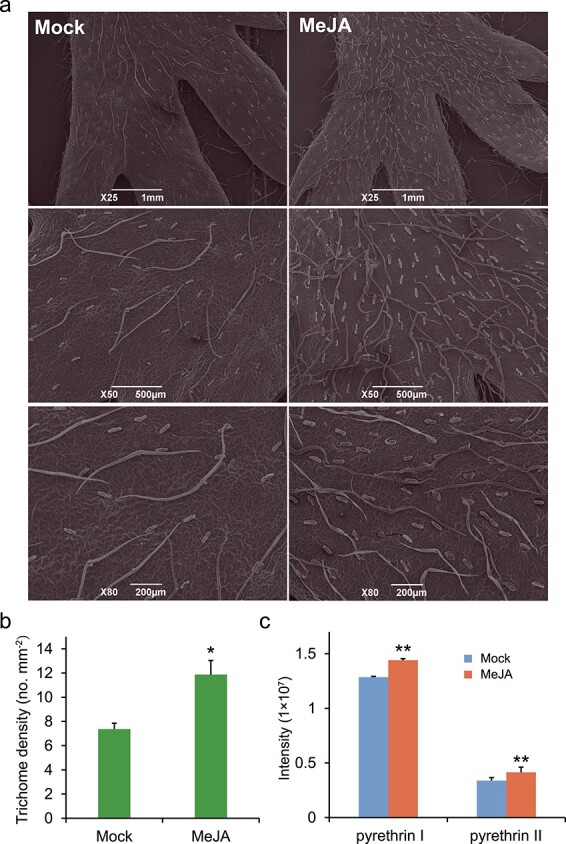
Prolonged MeJA treatment regulates trichomes density and pyrethrin content of *Tanacetum cinerariifolium* leaves. **a** Scanning electron micrographs of *T. cinerariifolium* leaves. Mock: seedlings treated with 0.8% ethanol; MeJA: seedlings treated with 300 μM MeJA dissolved in 0.8% ethanol. MeJA was applied every 2 d for 14 d. **b** Trichome number on *T. cinerariifolium* leaves. Bars and error bars indicate the mean ± SD from six biological replicates. **c** Relative content of total pyrethrins. Bars and error bars indicate the mean ± SD from four biological replicates. ^*^P < 0.05, ^**^P < 0.01 (Student’s paired *t-*test).

Phytohormones promote the formation of leaf trichomes in plants [31, 32]. In *T. cinerariifolium*, the ovary epidermis has a high density of glandular trichomes in which pyrethrin precursors are produced [33]. However, the trichome number on leaves is limited. To examine the effect of MeJA treatment on trichome development in *T. cinerariifolium*, seedlings at the four-leaf stage were subjected to continuous MeJA treatment for 2 weeks. A significant (1.6-fold) increase in trichome density was observed on MeJA-treated leaves compared with mock leaves (Fig. 7a and b), suggesting that prolonged application of MeJA promotes the initiation of glandular trichomes. Given that the short MeJA treatment only transiently enhanced the pyrethrin content in the first few hours, we performed high-performance liquid chromatography (HPLC) analysis to detect pyrethrins in samples treated with MeJA for 2 weeks. Compared with mock plants, the pyrethrin I and pyrethrin II contents were significantly increased in MeJA-treated plants by 14% at 2 d post treatment, in contrast to the rapid decline in pyrethrin content at 8 h after short-term MeJA treatment (Fig. 7c). Thus, prolonged application of MeJA positively regulated glandular trichome development and pyrethrin accumulation in leaves.

## Discussion

Jasmonates play an important role in controlling secondary metabolite synthesis through a network of TFs that regulate metabolite biosynthesis genes [11]. In this study, transient short-term MeJA treatment of *T. cinerariifolium* leaves strongly induced expression of upstream genes in the pyrethrin biosynthesis pathway, although the expression levels quickly declined. A transient increase in pyrethrin accumulation resulted, which lasted for only several hours and thereafter declined. The pyrethrin biosynthetic pathway is long and complex. Most pyrethrin biosynthesis genes and precursors are expressed in and exported from trichomes to synthesize pyrethrins, which mainly accumulate in the secretory cavity of the ovary wall to protect the seed [33]. Mature leaves contain no corresponding storage structures and the sudden excessive accumulation of pyrethrins might disturb the cellular homeostasis. This was shown previously in *T. cinerariifolium* with overexpression of *TcCHS* driven by the Chrysanthemum RUBISCO promoter. Overexpression of TcCHS increased the pyrethrin content but led to a significant decrease in chlorophyll content [34]. A pyrethrin content of 0.05% or 0.001% still exerts good control of pyrethrin-sensitive herbivorous insects [35, 36]. The average pyrethrin content in leaves is ~0.1% (dry weight), which is still toxic to pyrethrin-sensitive insects in leaves without external stimuli. We presume that it is not necessarily energetically costly to respond to JAs to increase the pyrethrin content.

Continuous MeJA treatment of seedlings at the four-leaf stage for 2 weeks increased the glandular trichome density and pyrethrin accumulation in the leaves. We speculate that under long-term MeJA exposure, seedlings gradually adapt to exogenous hormone signals, which results in morphological changes that enable increased pyrethrin production. These changes have been reported in other Asteraceae species, such as *Asteraceae annua*, which showed upregulation of the artemisinin content in leaves together with trichome development on the leaf surface [37]. Similarly, a significant correlation between the monoterpene and sesquiterpene content and gland density on leaves in chrysanthemum has been reported [38].

MYC, a member of the bHLH superfamily that harbors the conserved HLH domain, can bind to the G-box or E-box motifs in the promoters of target genes to regulate their involvement in secondary metabolite biosynthesis [39]. As expected, TcMYC2 could bind to the *TcCHS*, *TcAOC*, and *TcGLIP* promoters and positively regulate expression of downstream genes *in vitro.* Plants transiently silenced for *TcMYC2* expression showed significant downregulation of pyrethrin biosynthesis genes and dramatically reduced pyrethrin contents (Fig. 6d and e). Thus, TcMYC2 positively regulated pyrethrin biosynthesis in *T. cinerariifolium* leaves. In JA-treated *Arabidopsis* plants, MYC2 positively regulates the expression of flavonoid biosynthesis genes [40]. MYC2 orthologs of tobacco regulate nicotine biosynthesis [41]. These MYC2 proteins show the strongest binding activity to the G-box followed by the 5′-CACGTG-3′ sequence. Thus, similar mechanisms operate in the regulation of the biosynthesis of various secondary metabolites, indicating that MYC2-like genes have conserved functions in these species [42]. MYCs generally exhibit functional redundancy and weakening of the function of one MYC can be compensated by other MYCs. In *Arabidopsis*, MYC5 has functions redundant with other MYCs, which participate in root growth inhibition, leaf senescence, and plant defense against insect attack and pathogen infection regulated by JA [43]. In *Nicotiana tabacum*, *NtMYC2a* coordinates with *NtMYC2b* to positively regulate nicotine biosynthesis [44]. In the current study, the molecular phenotype of *TcMYC2*-silenced *T. cinerariifolium* plants was not rescued by other potential MYCs, suggesting that *TcMYC2* might be an indispensable, non-redundant positive regulator of pyrethrin biosynthesis in *T. cinerariifolium.*

Although TcMYC2 showed strong activity on pyrethrin biosynthesis genes *in vitro* and a strong decrease in pyrethrin content in VIGS plants, gene expression and pyrethrin content in leaves with transient overexpression of *TcMYC2* showed only a slight and noncontinuous increase. In other studies, MYC-mediated continuous activation of JA responses inhibit growth, and are potentially harmful or fatal [45]. For example, overexpression of *TwMYC2a* and *TwMYC2b* results in downregulation of triptolide biosynthesis in hairy roots of *Tripterygium wilfordii* [46]. A possible reason is that *TcMYC2* has multiple functions and may activate other negative transcription factors simultaneously. For instance, MYC2 directly binds to the promoter of JAZ repressors, which restrain JA response [47, 48]. Plants overexpressing *MYC2* constitutively resynthesize JAZ repressors in response to JA stimulation to close activation of the pathway in a controlled manner [48].

To further explore the function of *TcMYC2*, stable transformation of hairy roots with *TcMYC2* was conducted. The target genes *TcAOC* and *TcGLIP* were significantly upregulated, whereas *TcCHS* transcripts were not detected, and no pyrethrins were produced either with incubation with substrates. *TcCHS* is almost exclusively expressed in glands, but no pyrethrins and glands are produced by hairy roots of *T. cinerariifolium* [49]. Furthermore, tissue differentiation is a prerequisite for production of certain secondary metabolites [50]. Typical examples include some *Hypericum* species that yield hypericin, but their hairy roots are devoid of hypericin [51]. To clarify whether glands are essential for pyrethrin synthesis, the function of *TcMYC2* requires further study in other tissues and experimental systems.

**Figure 8:**
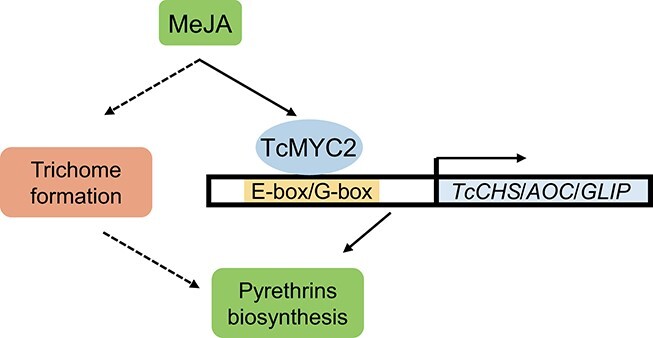
Working model for regulation of pyrethrin biosynthesis by TcMYC2 in response to methyl jasmonate (MeJA). Exposure to MeJA induces TcMYC2, which activates *TcCHS*, *TcAOC*, and *TcGLIP* by binding to their promoters and subsequently regulates pyrethrin biosynthesis. MeJA also regulates trichome density by an unknown mechanism.

In summary, this study demonstrates that MeJA induces *TcMYC2* expression in *T. cinerariifolium* and subsequently activates the expression of pyrethrin biosynthesis genes by binding to their promoters. Prolonged exposure of *T. cinerariifolium* seedlings to MeJA slightly enhances production of glandular trichomes, and consequently pyrethrin accumulation, but only in immature leaves (Fig. 8). This study reports the first of likely several TFs that are indispensable to promoting the expression of pyrethrin biosynthesis genes and pyrethrin accumulation. Elucidation of the full spectrum of relevant TFs may provide a novel means of enhancing pyrethrin yield through metabolic engineering of *T. cinerariifolium*.

## Materials and methods

### Plant materials

*T. cinerariifolium* ‘W99’ was used in this study because it is easy rooting, grows rapidly, and has high pyrethrin content. *T. cinerariifolium* is a self-incompatible, highly heterogenous species [52], and individual genotypes have a unique pyrethrin content. One-month-old uniformly sized ‘W99’ seedlings grown *in vitro* on half-strength Murashige and Skoog medium in large airtight containers of identical size were used for MeJA treatment. The seedlings were grown at 25°C under a 16 h/8 h (light/dark) photoperiod.

Pyrethrum flower heads and leaves at different developmental stages (S1–S6 [3] and L1–L2 [15], respectively,) as well as flower tissues, were collected in triplicate, immediately frozen in liquid nitrogen, and stored at −80°C until use.

### MeJA treatment

For short-term MeJA treatment, 1-month-old ‘W99’ seedlings grown *in vitro* were sprayed with 5 mL of 2 mM MeJA dissolved in 0.8% ethanol. Leaves were collected in triplicate after 0 (control), 2, 4, 6, 8, 12, and 24 h. The samples were immediately frozen in liquid nitrogen and stored at −80°C until use.

For long-term MeJA treatment, ‘W99’ seedlings at the four-leaf stage were sprayed with 5 mL of 300 μM MeJA dissolved in 0.8% ethanol, and the roots were irrigated with 5 mL every 2 d for a total of 2 weeks. Plants sprayed with 0.8% ethanol served as a mock control. Subsequently, leaves were collected and oven-dried at 50°C for 48 h to constant dry weight.

### RNA extraction, transcriptome assembly, and qRT-PCR

Total RNA was extracted from the samples using the standard phenol–chloroform extraction method. Twenty-four RNA-seq libraries were constructed and sequenced by a commercial sequencing service provider (Benagen, Wuhan, China). After filtering out low-quality reads and adapter sequences using fastp v0.18.0 software [53], the clean reads were *de novo* assembled using Trinity v2.6.6 software [54]. The assembly quality was evaluated using N50 and BUSCO v3.0.2 software [55]. Sequences were annotated with Diamond v0.9.2.24 using the nucleotide sequence (NT), nonredundant protein (NR), Clusters of Orthologous groups of proteins (COG), Kyoto Encyclopedia of Genes and Genomes (KEGG), Swiss-Prot, and Gene Ontology (GO) databases. Gene expression levels were calculated using the RNA-Seq by Expectation–Maximization v1.3.1 software [56] and normalized to the FPKM values [57]. The DEGs were identified using DESeq2 software [58] with the following thresholds: |log_2_ fold change| > 1; *P*_adj_ < 0.05. GO and KEGG enrichment analyses of the DEGs was performed using ClusterProfiler [59]. A WGCNA (v1.703) analysis was performed to identify modules of highly correlated genes [60]. KEGG and GO annotation of the modules was performed with KOBAS v3.0 software [61]. The protein association network was searched using the STRING v11.5 database (http://string-db.org/). Hub genes were screened using CytoHubba [62], and Cytoscape v3.8.2 was used for visual analysis [63].

The qRT-PCR assay was performed on the Roche LightCycler® 96 Real-time PCR System (Roche, Basel, Switzerland), in accordance with the manufacturer’s instructions, using SYBR Premix Ex Taq II (Takara, Kusatsu, Japan) and sequence-specific primers described previously [12]. Other primers were based on the transcriptome and checked using TBtools [64]. Primers used for qRT-PCR are listed in Table S1 (see online supplementary material). Three biological replicates were performed for each gene, with two technical repeats per biological replicate. *GADPH* was used as the internal reference gene. Relative gene expression was calculated using the 2^−ΔΔ*C*t^ method [65]. Student’s two-tailed *t*-test was used to determine statistical significance at the 5% and 1% significance levels.

### Transcription factor and *TcMYC2* identification

PlantTFDB 5.0 was used to predict all TFs [66]. Protein domains were identified using the SMART online tool [67]. After filtering out weakly expressed genes and proteins lacking conserved domains, all bHLH TFs were visualized. The FPKM values of genes from the MeJA treatment and flower stage transcriptome were used to perform a cluster analysis. Heatmaps were generated using TBtools [64].

The open reading frame of *TcMYC2* was cloned from *T. cinerariifolium* cDNA using sequence-specific primers (Table S1, see online supplementary material). Sequences of *T. cinerariifolium* genes homologous to Arabidopsis genes were identified by a BLAST search against The Arabidopsis Information Resource database, and to genes of other plant species by a BLAST search against the National Center for Biotechnology Information database. The AtMYC proteins were scanned using HmmerSearch v3.0 software with the PfamA domain database [68]. TcMYC2 and AtMYC family coding sequences were aligned using mafft v7.158 software [69]. A maximum-likelihood phylogenetic tree was constructed using IQtree2 software [70], using the best-fit model (JTT + F + I + G4) selected by ModelFinder [71] and with 1000 bootstrap replications.

### Cloning and bioinformatic analysis of gene promoters

The promoter sequences of *TcCHS*, *TcALDH*, *TcAOS*, and *TcGLIP* were mined from the draft genome sequence of *T. cinerariifolium* [72]. The associated *cis*-elements and their biological functions were determined using PlantCARE [73], and the *cis*-regulatory elements (E-box, G-box, and G-box-like) were identified using PlantPAN3.0 [74].

### Y1H assay

The full-length *TcMYC2* cDNA was cloned into the pGADT7 vector using *Nde*I and *Xho*I restriction endonucleases. The promoter fragments of *TcCHS*, *TcGLIP*, *TcALDH*, and *TcAOC* were cloned into the *Eco*R1- and *Mlu*I-digested pHis2.1 vector. The resultant pGADT7:*TcMYC2* plasmid was co-transformed into yeast strain Y187 together with pHis2.1:*TcCHS*, pHis2.1:*TcGLIP*, pHis2.1:*TcAOC*, or pHis2.1:*TcALDH* using the Super Yeast Transformation Kit (Coolaber, Beijing, China). The transformed yeast cells were selected on DDO (SD/−Leu/−Trp) medium and interactions were tested on TDO (SD/−Leu/−Trp/−His) medium at 30°C for 3 d.

### Dual-LUC transient expression assay

To generate reporter constructs, promoter fragments of downstream genes (*TcCHS*, *TcGLIP*, *TcALDH*, and *TcAOC*) were cloned into the *Hin*dIII-linearized pGreenll0800-LUC vector. The *TcMYC2* coding sequence was cloned into the *Hin*dIII-linearized pGreenII62-SK vector under the control of the CaMV *35S* promoter. The resultant vectors were transiently coexpressed in *N. benthamiana* leaves. Luminescence was detected using the LB 985 Nightshade system (Berthold, Bad Wildbad, Germany). Introduction of the pGreen-62-SK empty vector and of pGreen-62-SK/pGreenII-0800-LUC:*proTcCHS*/*TcGLIP*/*TcAOC* constructs served as a negative control.

### EMSA

The open reading frame without the terminator codon of *TcMYC2* was inserted in the pET6HN-C vector protein, fused in the N-terminal frame of 6 × His. The vector pET6HN-C-*TcMYC2*–6 × His was transformed into *Escherichia coli* strain Rosetta (DE3). For induction of the recombinant protein, 0.5 mM IPTG was used, and the cultures were incubated at 18°C for 16 h. Then, Ni-NTA was used to purify the recombinant proteins.

For EMSA, the promoter fragments of *TcCHS*, *TcAOC*, and *TcGLIP* contained E-box or G-box *cis*-elements. FAM-labeled probes and the same unlabeled DNA fragments, *cis*-element mutant DNA fragments were used as competitors in the assay. The EMSA assays were performed as described previously [75]. FAM-labeled DNA was detected by the chemiluminescence method.

### Subcellular localization of *TcMYC2*

The coding sequence of *TcMYC2* was amplified by PCR using sequence-specific primers containing the *Swa*I and *Kpn*I restriction sites at their ends (Table S1, see online supplementary material). The PCR product was cloned into pSuper1300-GFP, derived from pCambia1300, using the ClonExpress® II One Step Cloning Kit (Vazyme Biotech, Nanjing, China). The resultant plasmid was introduced into *Agrobacterium tumefaciens* strain GV3101, and then co-infiltrated into *N. benthamiana* leaves together with the *RFP-NLS* plasmid (nuclear marker) following methods described previously [76]. The empty vector was used as a control. After 72 h of weak light exposure, fluorescence signals were observed with a confocal laser scanning microscope (Leica TCS-SP8; Leica, Wetzlar, Germany).

### *TcMYC2* stable transformation of hairy roots

The full-length *TcMYC2* coding sequence was inserted into pBI121 vector digested with *Bam*HI and *Sac*I using homologous recombination, and transferred into *A. rhizogenes* strain MSU440. The infection process followed that of previous studies [49].

Genomic DNA was extracted from transgenic hairy roots after 30 d using the CTAB method. Successful transfer of the transgene-positive cells was confirmed by PCR. The primers for *rolB* of the *A. rhizogenes* Ri plasmid were designed in accordance with a previous study [77]. Transgenic hairy roots were determined by qRT-PCR and GC–MS analysis.

### Transient overexpression of *TcMYC2* in *T. cinerariifolium* leaves

The full-length *TcMYC2* coding sequence was cloned into the *Hin*dIII-linearized pGreenII62-SK vector downstream of the CaMV *35S* promoter using gene-specific primers (Table S1, see online supplementary material). The pGreenII62-SK: *TcMYC2* vector was co-transformed together with the helper plasmid pSoup19 into *A. tumefaciens* strain GV3101. Leaves of *T. cinerariifolium* were placed in a 500 mL beaker containing *A. tumefaciens* suspension. The leaves were infiltrated under vacuum of 0.23 atm for 5 min. The agroinfiltrated leaves were dried with filter paper and stored in a Petri dish lined with a moisture-retaining filter paper at the bottom, as described previously [78]. After 2, 4, and 6 d of incubation, the agroinfiltrated leaf samples were used for qRT-PCR, and the 4 d samples were used for HPLC analysis.

### VIGS assay

Tobacco rattle virus (TRV)-based vectors, pTRV1 and pTRV2, were used for the VIGS assay. A 256 bp fragment complementary to the *TcMYC2* coding sequence was designed, searched in the local transcriptome library using siRNA Finder [79], and cloned into the *Bam*HI-linearized pTRV2 vector by homologous recombination using gene-specific primers (Table S1, see online supplementary material). The pTRV2:*TcMYC2* and pTRV1 plasmids were transformed into chemically active GV3101 cells using the liquid nitrogen freeze–thaw method. The VIGS assay was conducted as described previously [80] with three biological replicates. Empty vectors were used as controls. After 14 d, the samples were subjected to qRT-PCR and HPLC analyses.

### Measurement of pyrethrin content by GC–MS

Leaf samples exposed to short-term MeJA treatment were ground into a fine powder in liquid nitrogen. Powdered tissue (0.5 g) was transferred to a tube containing 2 mL methyl tert–butyl ether (MTBE), with 0.01 ng/mL tetradecane as an internal standard. The tube was vortexed for 3 min at maximum speed and incubated at 24°C with rotation of 50 rpm for 2 h. The sample was dried using Na_2_SO_4_ and filtered through a 0.22-μm mesh filter. The pyrethrin content was measured using a GC/MS-QP2010Ultra apparatus (Shimadzu Corporation, Kyoto, Japan) equipped with a HP-5 MS column. Three biological replicates were performed for each sample. The GC process was performed as described previously [81]. Commercial pyrethrum extract (Sigma-Aldrich, St Louis, MI, USA) was used as the standard.

### Pyrethrin content measurement by HPLC

The transiently transformed samples and samples exposed to long-term MeJA treatment were oven-dried at 50°C for 48 h to constant dry weight. The dried leaves were milled into a fine powder and dissolved in *n*-hexane at a concentration of 1 g per 6 mL in glass tubes with screwcaps. After vortexing for 30 s, the samples were extracted in an ultrasound water bath for 10 min, then vortexed again for 30 s, as described previously [74]. The samples were filtered through a 0.22-μm mesh filter. The pyrethrin content was measured by HPLC, with three biological replicates, as described previously [6]. Commercial pyrethrum extract (Sigma-Aldrich, St Louis, MI, USA) was used as the standard.

### Analysis of the effect of MeJA on trichome density

Seedlings at the four-leaf stage were sprayed with 300 μM MeJA every 2 d for 2 weeks. The mock control was treated with 0.8% ethanol. The sixth leaf, which emerged after MeJA treatment, was excised and prepared for scanning electron microscopy as described previously [82]. The trichome number was determined from the micrographs of six biological replicates. Statistical significance was determined by Student’s *t*-test at *P* < 0.05.

## Acknowledgements

This work was supported by the National Natural Science Foundation of China (31902051, 32160718) and China Postdoctoral Science Foundation (2018 M640720), National Key Research and Development Project (2019YFD1001500), Fundamental Research Funds for the Central Universities (2662019FW016), Natural Science Research Project of Guizhou (KY[2022]170, ZK[2022]301) The authors also thank She-liang Wang, College of Resources & Environment at Huazhong Agricultural University for assistance.

## Author contributions

T.Z., H.H., J-W.L., and C-Y.W . planned and designed research; T.Z., J-W.L., Z-Z.X., L.Z., Q.Y., and H.H . performed research; T.Z., J-W.L., and H.H . analysed data; H.H., T.Z., J-W.L., and C-Y.W . wrote the paper; H.H., T.Z., J-W.L., J-J.L., J.L., Z-L. C., M.A.J. and C-Y .W . revised the paper.

## Data availability

All transcriptome raw data can be downloaded from NCBI (BioProjects: PRJNA723714, https://www.ncbi.nlm.nih.gov/sra/PRJNA723714).

## Conflict of interests

The authors declare that they have no conflict of interest.

## Supplementary data

Supplementary data is available at *Horticulture Research * online.

## Supplementary Material

supp_data_uhac178

## References

[1] Casida JE , Quistad GB. Pyrethrum Flowers: Production, Chemistry, Toxicology, and Uses. Oxford: Oxford University Press; 1995.

[2] Katsuda Y . Development of and future prospects for pyrethroid chemistry. *Pestic Sci*. 1999;55:775–82.

[3] Ramirez AM . Pyrethrum secondary metabolism: biosynthesis, localization and ecology of defence compoundsVol. Ph.D. Thesis, Wageningen University, 2013.

[4] Lybrand DB , Xu H, Last RL et al. How plants synthesize pyrethrins: safe and biodegradable insecticides. *Trends Plant Sci*. 2020;25:1240–51. 32690362 10.1016/j.tplants.2020.06.012 PMC7677217

[5] Rivera SB , Swedlund BD, King GJ et al. Chrysanthemyl diphosphate synthase: isolation of the gene and characterization of the recombinant non-head-to-tail monoterpene synthase from *chrysanthemum cinerariaefolium*. *P Natl Acad Sci USA*. 2001;98:4373–8. 10.1073/pnas.071543598 PMC31842 11287653

[6] Hu H , Li J, Delatte T et al. Modification of chrysanthemum odour and taste with chrysanthemol synthase induces strong dual resistance against cotton aphids. *Plant Biotechnol J*. 2018;16:1434–45. 29331089 10.1111/pbi.12885 PMC6041446

[7] Xu H , Moghe GD, Wiegert-Rininger K et al. Coexpression analysis identifies two oxidoreductases involved in the biosynthesis of the monoterpene acid moiety of natural pyrethrin insecticides in *Tanacetum cinerariifolium*. *Plant Physiol*. 2018;176:524–37. 29122986 10.1104/pp.17.01330 PMC5761793

[8] Xu H , Li W, Schilmiller AL et al. Pyrethric acid of natural pyrethrin insecticide: complete pathway elucidation and reconstitution in *Nicotiana benthamiana*. *New Phytol*. 2019;223:751–65. 30920667 10.1111/nph.15821

[9] Kikuta Y , Ueda H, Takahashi M et al. Identification and characterization of a GDSL lipase-like protein that catalyzes the ester-forming reaction for pyrethrin biosynthesis in *Tanacetum cinerariifolium*- a new target for plant protection. *Plant J*. 2012;71:183–93. 22385412 10.1111/j.1365-313X.2012.04980.x

[10] Ghorbel M , Brini F, Sharma A et al. Role of jasmonic acid in plants: the molecular point of view. *Plant Cell Rep*. 2021;40:1471–94. 33821356 10.1007/s00299-021-02687-4

[11] Wasternack C , Strnad M. Jasmonates are signals in the biosynthesis of secondary metabolites — pathways, transcription factors and applied aspects — a brief review. *New Biotechnol*. 2019;48:1–11. 10.1016/j.nbt.2017.09.007 29017819

[12] Li W , Zhou F, Pichersky E. Jasmone hydroxylase, a key enzyme in the synthesis of the alcohol moiety of pyrethrin insecticides. *Plant Physiol*. 2018;177:1498–509. 29967096 10.1104/pp.18.00748 PMC6084660

[13] Li W , Lybrand DB, Zhou F et al. Pyrethrin biosynthesis: the cytochrome P450 oxidoreductase CYP82Q3 converts jasmolone to pyrethrolone. *Plant Physiol*. 2019;181:934–44. 31451551 10.1104/pp.19.00499 PMC6836846

[14] Yang T , Stoopen G, Wiegers G et al. Pyrethrins protect pyrethrum leaves against attack by Western flower Thrips. *J Chem Ecol*. 2012;38:370–7. 22456949 10.1007/s10886-012-0097-7 PMC3324680

[15] Zito SW , Zieg RG, Staba EJ. Distribution of pyrethrins in oil glands and leaf tissue of *chrysanthemum cinerariaefolium*. *Planta Med*. 1983;47:205–7. 17404915 10.1055/s-2007-969986

[16] Baldwin IT , Karb MJ, Callahan P. Foliar and floral pyrethrins of *chrysanthemum cinerariaefolium* are not induced by leaf damage. *J Chem Ecol*. 1993;19:2081–7. 24249382 10.1007/BF00983810

[17] Li L , Wang D, Zhou L et al. JA-responsive transcription factor SmMYB97 promotes phenolic acid and tanshinone accumulation in *salvia miltiorrhiza*. *J Agr Food Chem*. 2020;68:14850–62. 33284615 10.1021/acs.jafc.0c05902

[18] Fu X , Peng B, Hassani D et al. AaWRKY9 contributes to light- and jasmonate-mediated to regulate the biosynthesis of artemisinin in *Artemisia annua*. *New Phytol*. 2021;231:1858–74. 33973259 10.1111/nph.17453

[19] Ma Y-N , Xu DB, Yan X et al. Jasmonate- and abscisic acid-activated AaGSW1-AaTCP15/AaORA transcriptional cascade promotes artemisinin biosynthesis in *Artemisia annua*. *Plant Biotechnol J*. 2021;19:1412–28. 33539631 10.1111/pbi.13561 PMC8313134

[20] Gao WJ , Meng Q, Luo H et al. Transcriptional responses for biosynthesis of flavor volatiles in methyl jasmonate-treated *Chrysanthemum indicum* var. aromaticum leaves. *Ind Crop Prod*. 2020;147:112254.

[21] An C , Sheng L, du X et al. Overexpression of CmMYB15 provides chrysanthemum resistance to aphids by regulating the biosynthesis of lignin. *Hortic Res*. 2019;6:84. 31645945 10.1038/s41438-019-0166-y PMC6804602

[22] Zhang W , Gao T, Li P et al. Chrysanthemum CmWRKY53 negatively regulates the resistance of chrysanthemum to the aphid *Macrosiphoniella sanborni*. *Hortic Res*. 2020;7:109. 32637137 10.1038/s41438-020-0334-0 PMC7327015

[23] Bai JF , Wang YK, Guo LP et al. Genomic identification and characterization of MYC family genes in wheat (*Triticum aestivum* L.). *BMC Genomics*. 2019;20:1032. 31888472 10.1186/s12864-019-6373-y PMC6937671

[24] Shen Q , Lu X, Yan T et al. The jasmonate-responsive AaMYC2 transcription factor positively regulates artemisinin biosynthesis in *Artemisia annua*. *New Phytol*. 2016;210:1269–81. 26864531 10.1111/nph.13874

[25] Hong GJ , Xue XY, Mao YB et al. *Arabidopsis* MYC2 interacts with DELLA proteins in regulating sesquiterpene synthase gene expression. *Plant Cell*. 2012;24:2635–48. 22669881 10.1105/tpc.112.098749 PMC3406894

[26] Xu YH , Liao YC, Lv FF et al. Transcription factor AsMYC2 controls the Jasmonate-responsive expression of ASS1 regulating Sesquiterpene biosynthesis in Aquilaria sinensis (lour.) Gilg. *Plant Cell Physiol*. 2017;58:1924–33. 29016977 10.1093/pcp/pcx122

[27] Zhang M , Jin X, Chen Y et al. TcMYC2a, a basic helix loop helix transcription factor, transduces ja-signals and regulates taxol biosynthesis in *Taxus chinensis*. *Front Plant Sci*. 2018;9:863. 29977250 10.3389/fpls.2018.00863 PMC6021540

[28] Dabiri M , Majdi M, Bahramnejad B. Partial sequence isolation of DXS and AOS genes and gene expression analysis of terpenoids and pyrethrin biosynthetic pathway of *chrysanthemum cinerariaefolium* under abiotic elicitation. *Acta Physiol Plant*. 2020;42:30.

[29] Kikuta Y , Ueda H, Nakayama K et al. Specific regulation of pyrethrin biosynthesis in *chrysanthemum cinerariaefolium* by a blend of volatiles emitted from artificially damaged conspecific plants. *Plant Cell Physiol*. 2011;52:588–96. 21296762 10.1093/pcp/pcr017

[30] Sultana S , Hu H, Gao L et al. Molecular cloning and characterization of the trichome specific chrysanthemyl diphosphate/chrysanthemol synthase promoter from *Tanacetum cinerariifolium*. *Sci Hortic*. 2015;185:193–9.

[31] Maes L , van Nieuwerburgh FCW, Zhang Y et al. Dissection of the phytohormonal regulation of trichome formation and biosynthesis of the antimalarial compound artemisinin in *Artemisia annua* plants. *New Phytol*. 2011;189:176–89. 20874804 10.1111/j.1469-8137.2010.03466.x

[32] Boughton AJ , Hoover K, Felton GW. Methyl jasmonate application induces increased densities of glandular trichomes on tomato. *J Chem Ecol*. 2005;31:2211–6. 16132222 10.1007/s10886-005-6228-7

[33] Ramirez AM , Stoopen G, Menzel TR et al. Bidirectional secretions from glandular trichomes of pyrethrum enable immunization of seedlings. *Plant Cell*. 2012;24:4252–65. 23104830 10.1105/tpc.112.105031 PMC3517248

[34] Li J , Xu Z, Zeng T et al. Overexpression of TcCHS increases Pyrethrin content when using a genotype-independent transformation system in pyrethrum (*Tanacetum cinerariifolium*). *Plan Theory*. 2022;11:1575. 10.3390/plants11121575 PMC9229838 35736726

[35] Andreev R , Kutinkova H, Baltas K. Non-chemical control of some important pests of sweet cherry. *Journal of Plant Protection Research*. 2008;48:503–8.

[36] Shukla S , Tiwari SK. The influence of pyrethrum extract on the developmental stages of the rice-moth, *Corcyra cephalonica* Stainton (Lepidoptera: Pyralidae). *Egypt J Biol*. 2012;14:57–62.

[37] Xie L , Yan T, Li L et al. An HD-ZIP-MYB complex regulates glandular secretory trichome initiation in *Artemisia annua*. *New Phytol*. 2021;231:2050–64. 34043829 10.1111/nph.17514

[38] Guan Y , Chen S, Chen F et al. Exploring the relationship between Trichome and Terpene chemistry in chrysanthemum. *Plan Theory*. 2022;11:1410. 10.3390/plants11111410 PMC9182802 35684184

[39] Hao Y , Zong X, Ren P et al. Basic helix-loop-helix (bHLH) transcription factors regulate a wide range of functions in *Arabidopsis*. *Int J Mol Sci*. 2021;22:7152. 34281206 10.3390/ijms22137152 PMC8267941

[40] Dombrecht B , Xue GP, Sprague SJ et al. MYC2 differentially modulates diverse jasmonate-dependent functions in *Arabidopsis*. *Plant Cell*. 2007;19:2225–45. 17616737 10.1105/tpc.106.048017 PMC1955694

[41] Todd AT , Liu E, Polvi SL et al. A functional genomics screen identifies diverse transcription factors that regulate alkaloid biosynthesis in *Nicotiana benthamiana*. *Plant J*. 2010;62:589–600. 20202168 10.1111/j.1365-313X.2010.04186.x

[42] Shoji T , Hashimoto T. Tobacco MYC2 regulates jasmonate-inducible nicotine biosynthesis genes directly and by way of the NIC2-locus ERF genes. *Plant Cell Physiol.* 2011;52:1117–30. 21576194 10.1093/pcp/pcr063

[43] Song S , Huang H, Wang J et al. MYC5 is involved in jasmonate-regulated plant growth, leaf senescence and defense responses. *Plant Cell Physiol*. 2017;58:1752–63. 29017003 10.1093/pcp/pcx112

[44] Zhang HB , Bokowiec MT, Rushton PJ et al. Tobacco transcription factors NtMYC2a and NtMYC2b form nuclear complexes with the ntjaz1 repressor and regulate multiple jasmonate-inducible steps in nicotine biosynthesis. *Mol Plant*. 2012;5:73–84. 21746701 10.1093/mp/ssr056

[45] Chico JM , Lechner E, Fernandez-Barbero G et al. CUL3BPM E3 ubiquitin ligases regulate MYC2, MYC3, and MYC4 stability and JA responses. *Proc Natl Acad Sci USA*. 2020;117:6205–15. 32123086 10.1073/pnas.1912199117 PMC7084108

[46] Huo YB , Zhang J, Zhang B et al. MYC2 transcription factors TwMYC2a and TwMYC2b negatively regulate Triptolide biosynthesis in *Tripterygium wilfordii* hairy roots. *Plants*. 2021;10:679. 33916111 10.3390/plants10040679 PMC8067133

[47] Figueroa P , Browse J. The *Arabidopsis* JAZ2 promoter contains a G-box and thymidine-rich module that are necessary and sufficient for jasmonate-dependent activation by MYC transcription factors and repression by JAZ proteins. *Plant Cell Physiol*. 2012;53:330–43. 22173100 10.1093/pcp/pcr178

[48] Chini A , Fonseca S, Fernández G et al. The JAZ family of repressors is the missing link in jasmonate signalling. *Nature*. 2007;448:666–71. 17637675 10.1038/nature06006

[49] Li JW , Zeng T, Xu ZZ et al. Ribozyme-mediated CRISPR/Cas9 gene editing in pyrethrum (*Tanacetum cinerariifolium*) hairy roots using a RNA polymerase II-dependent promoter. Plant Methods. 2022;18:32. 10.1186/s13007-022-00863-5 PMC8925089 35292048

[50] Ramawat KG . An introduction to the process of cell, tissue, and organ differentiation, and production of secondary metabolites. In: Ramawat KG (ed). *Plant Cell and Tissue Differentiation and Secondary Metabolites*. Springer International Publishing, Cham, 2021, pp. 1–22.

[51] Nigutová K , Kusari S, Sezgin S et al. Chemometric evaluation of hypericin and related phytochemicals in 17 in vitro cultured *Hypericum* species, hairy root cultures and hairy root-derived transgenic plants. *J Pharm Pharmacol*. 2019;71:46–57. 28722156 10.1111/jphp.12782

[52] Brewer JG . Incompatibility relationship in pyrethrum (*chrysanthemum cinerariaefolium* Vis). *Euphytica*. 1974;23:45–7.

[53] Chen SF , Zhou YQ, Chen YR et al. Fastp: an ultra-fast all-in-one FASTQ preprocessor. *Bioinformatics*. 2018;34:i884–90. 30423086 10.1093/bioinformatics/bty560 PMC6129281

[54] Grabherr MG , Haas BJ, Yassour M et al. Full-length transcriptome assembly from RNA-Seq data without a reference genome. *Nat Biotechnol.* 2011;29:644–52. 21572440 10.1038/nbt.1883 PMC3571712

[55] Simão FA , Waterhouse RM, Ioannidis P et al. BUSCO: assessing genome assembly and annotation completeness with single-copy orthologs. *Bioinformatics*. 2015;31:3210–2. 26059717 10.1093/bioinformatics/btv351

[56] Li B , Dewey CN. RSEM: accurate transcript quantification from RNA-Seq data with or without a reference genome. *BMC Bioinformatics*. 2011;12:16 323. 21816040 10.1186/1471-2105-12-323 PMC3163565

[57] Trapnell C , Williams BA, Pertea G et al. Transcript assembly and quantification by RNA-Seq reveals unannotated transcripts and isoform switching during cell differentiation. *Nat Biotechnol*. 2010;28:511–5. 20436464 10.1038/nbt.1621 PMC3146043

[58] Anders S , Huber W. Differential expression analysis for sequence count data. *Genome Biol*. 2010;11:R106. 20979621 10.1186/gb-2010-11-10-r106 PMC3218662

[59] Yu G , Wang LG, Han Y et al. Cluster profiler: an R package for comparing biological themes among gene clusters. *OMICS J Integr Biol*. 2012;16:284–7. 10.1089/omi.2011.0118 PMC3339379 22455463

[60] Zhang B , Horvath S. A general framework for weighted gene co-expression network analysis. *Stat Appl Genet Mol Biol*. 2005;4:Article17. 16646834 10.2202/1544-6115.1128

[61] Bu D , Luo H, Huo P et al. KOBAS-i: intelligent prioritization and exploratory visualization of biological functions for gene enrichment analysis. *Nucleic Acids Res*. 2021;49:W317–25. 34086934 10.1093/nar/gkab447 PMC8265193

[62] Chin CH , Chen SH, Wu HH et al. cytoHubba: identifying hub objects and sub-networks from complex interactome. *BMC Syst Biol*. 2014;8:S11. 25521941 10.1186/1752-0509-8-S4-S11 PMC4290687

[63] Shannon P , Markiel A, Ozier O et al. Cytoscape: a software environment for integrated models of biomolecular interaction networks. *Genome Res*. 2003;13:2498–504. 14597658 10.1101/gr.1239303 PMC403769

[64] Chen CJ , Chen H, Zhang Y et al. TBtools: an integrative toolkit developed for interactive analyses of big biological data. *Mol Plant*. 2020;13:1194–202. 32585190 10.1016/j.molp.2020.06.009

[65] Livak KJ , Schmittgen TD. Analysis of relative gene expression data using real-time quantitative PCR and the 2− ΔΔCT method. *Methods*. 2001;25:402–8. 11846609 10.1006/meth.2001.1262

[66] Tian F , Yang DC, Meng YQ et al. PlantRegMap: charting functional regulatory maps in plants. *Nucleic Acids Res*. 2019;48:D1104–13. 10.1093/nar/gkz1020 PMC7145545 31701126

[67] Letunic I , Khedkar S, Bork P. SMART: recent updates, new developments and status in 2020. *Nucleic Acids Res*. 2021;49:D458–60. 33104802 10.1093/nar/gkaa937 PMC7778883

[68] Potter SC , Luciani A, Eddy SR et al. HMMER web server: 2018 update. *Nucleic Acids Res*. 2018;46:W200–w204. 29905871 10.1093/nar/gky448 PMC6030962

[69] Katoh K , Standley DM. MAFFT multiple sequence alignment software version 7: improvements in performance and usability. *Mol Biol Evol*. 2013;30:772–80. 23329690 10.1093/molbev/mst010 PMC3603318

[70] Minh BQ , Schmidt HA, Chernomor O et al. IQ-TREE 2: new models and efficient methods for phylogenetic inference in the genomic era. *Mol Biol Evol*. 2020;37:1530–4. 32011700 10.1093/molbev/msaa015 PMC7182206

[71] Kalyaanamoorthy S , Minh BQ, Wong TKF et al. ModelFinder: fast model selection for accurate phylogenetic estimates. *Nat Methods*. 2017;14:587–9. 28481363 10.1038/nmeth.4285 PMC5453245

[72] Yamashiro T , Shiraishi A, Satake H et al. Draft genome of *Tanacetum cinerariifolium*, the natural source of mosquito coil. *Sci Rep*. 2019;9:18249–9. 31796833 10.1038/s41598-019-54815-6 PMC6890757

[73] Lescot M , Déhais P, Thijs G et al. PlantCARE, a database of plant cis-acting regulatory elements and a portal to tools for in silico analysis of promoter sequences. *Nucleic Acids Res*. 2002;30:325–7. 11752327 10.1093/nar/30.1.325 PMC99092

[74] Chow CN , Lee TY, Hung YC et al. PlantPAN3.0: a new and updated resource for reconstructing transcriptional regulatory networks from ChIP-seq experiments in plants. *Nucleic Acids Res*. 2019;47:D1155–63. 30395277 10.1093/nar/gky1081 PMC6323957

[75] Xu H , Luo D, Zhang F. DcWRKY75 promotes ethylene induced petal senescence in carnation (*Dianthus caryophyllus* L.). *Plant J*. 2021;108:1473–92. 34587330 10.1111/tpj.15523

[76] Luo J , Wang H, Chen S et al. CmNAC73 mediates the formation of green color in chrysanthemum flowers by directly activating the expression of chlorophyll biosynthesis *HEMA1* and *CRD1*. *Genes*. 2021;12:704. 34066887 10.3390/genes12050704 PMC8151904

[77] Xiao X , Ma F, Chen C et al. High efficient transformation of auxin reporter gene into trifoliate orange via *agrobacterium rhizogenes*-mediated co-transformation. *Plant Cell Tissue Organ Cult*. 2014;118:137–46.

[78] Jung S-K , McDonald KA, Dandekar AM. Effect of leaf incubation temperature profiles on *agrobacterium tumefaciens*-mediated transient expression. *Biotechnol Prog*. 2015;31:783–90. 25829353 10.1002/btpr.2077

[79] Lück S , Kreszies T, Strickert M et al. siRNA-finder (si-fi) software for RNAi-target design and off-target prediction. *Front Plant Sci*. 2019;10:1023. 31475020 10.3389/fpls.2019.01023 PMC6704232

[80] Senthil-Kumar M , Mysore KS. Tobacco rattle virus-based virus-induced gene silencing in *Nicotiana benthamiana*. *Nat Protoc*. 2014;9:1549–62. 24901739 10.1038/nprot.2014.092

[81] Zeng T , Li JW, Zhou L et al. Transcriptional responses and GCMS analysis for the biosynthesis of pyrethrins and volatile terpenes in *Tanacetum coccineum*. *Int J Mol Sci*. 2021;22:13005. 34884809 10.3390/ijms222313005 PMC8657971

[82] Li JJ , Hu H, Chen Y et al. Tissue specificity of (E)-β-farnesene and germacrene D accumulation in pyrethrum flowers. *Phytochemistry*. 2021;187:112768. 33932787 10.1016/j.phytochem.2021.112768

