# Supplementary material for: TcMYC2 regulates Pyrethrin biosynthesis in Tanacetum cinerariifolium
Source: Hortic Res. 2022 Aug 24;9:uhac178. doi: 10.1093/hr/uhac178 (PMC9627524; doi:10.1093/hr/uhac178)
Supplement: supp_data_uhac178 [file supp_data_uhac178.zip › Supplemental information.docx]

**Supplemental information**

**Supplemental Results**

**Transcriptome data assembly and analysis**

40,694,832 Raw reads were obtained from each library. After quality control, at least 39,584,230 clean reads and 5.94 G clean bases were obtained from each library. The percentage of clean reads, Q30 were all over 96.23% and 91.88%, respectively. The average GC content was 42.49%. Finally, 94,591 unigenes were obtained. The average length, N50, and GC contents of the unigene were 975 bp,1655 bp, and 37.32%, respectively (Fig. S1a). BUSCO analysis indicates that most genes are present in the assembly and sequencing data were of sufficient quality (Fig S1b). 41,025 Unigenes annotated on Nr database (43.37%), 25,637 annotated on Pfam database (27.10%), 24,910 annotated on Uniprot database (26.33%), and 24,337 annotated on GO database (25.73%). A total of 20,317 (21.48%) were annotated on eggNOG database, 11,574 (12.24%) were annotated on KEGG database, and 7,416 (7.84%) were annotated on KEGG pathway database (Fig S1c), and based on the annotation result of NR database, the distribution map was drawn by the ratios of different species (Fig S1d), the top 5 species annotated are *Artemisia annua* (78.26%), *Helianthus annuus* (6.24%), and *Cynara cardunculus* var. scolymus (4.57%). *Lactuca sativa* (3.46%), *Chrysanthum morifolium* (0.56%). All of the samples were checked by the Pearson correlation analysis and Principal Component Analysis (PCA), indicating high consistency among the replicates (Fig S2a and b). Cluster analysis was conducted according to the expression of genes in the samples and indicated significant differences between untreated and treated groups (Fig S2c). Gene expression was determined by FPKM (Fragments Per kb of transcript per Million mapped fragments) method. P-adj<0.05 and |log_2_FoldChange| >1 were used as the criteria for screening differentially expressed genes (DEGs). The results suggest that DEGs occur mainly in the early stage of treatment (Fig S2d).

**Supplemental Figures**


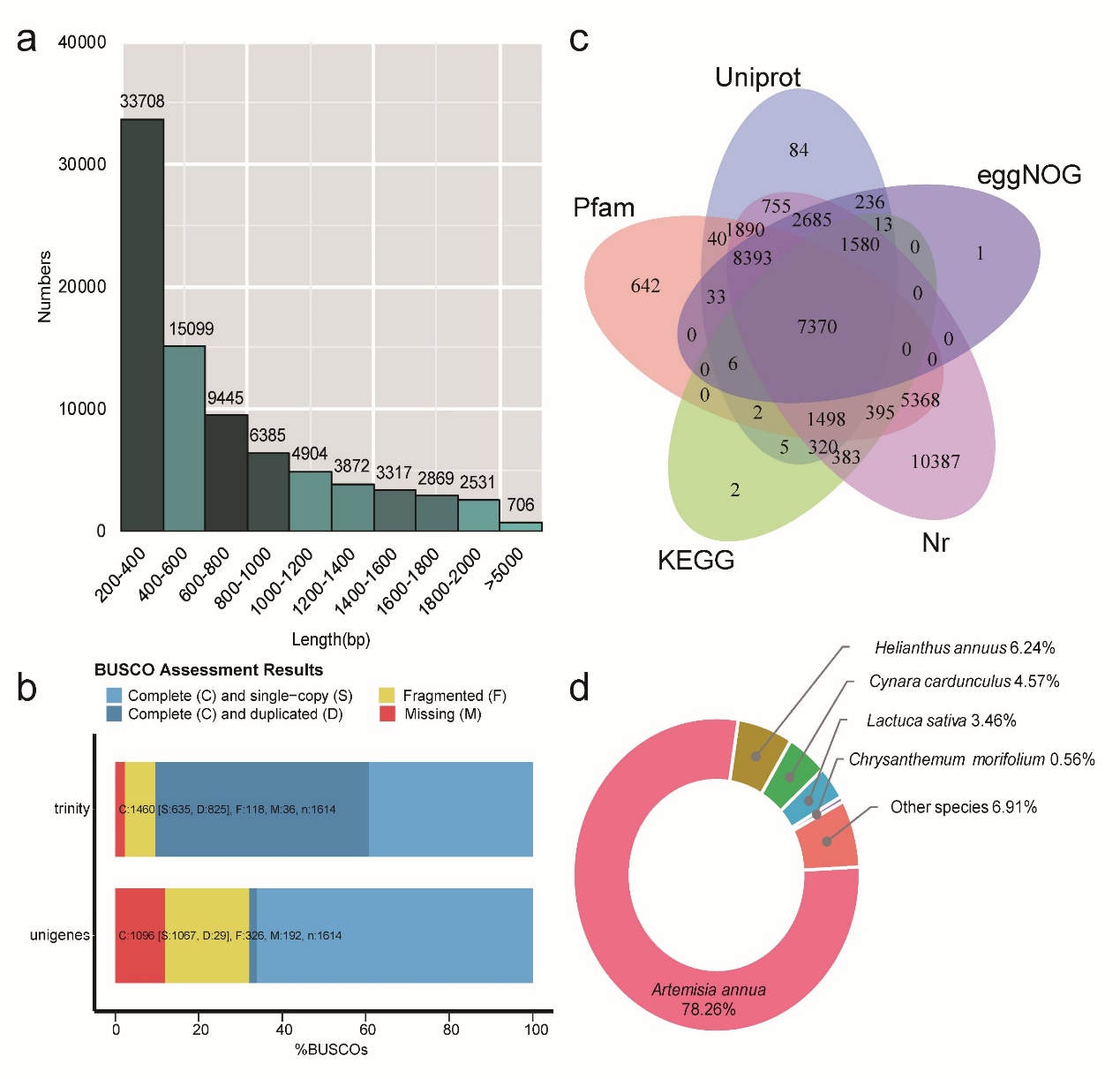


**Figure S1.** Transcriptome assembly and gene functional annotation. **a** Length distribution of the assembled unigenes. **b** BUSCO assessment results. **c** Total number of functional annotations in the Nt, Nr, KOG, GO, and Pfam databases. **d** Species distribution in the results of a BLASTX search against the Nr database.


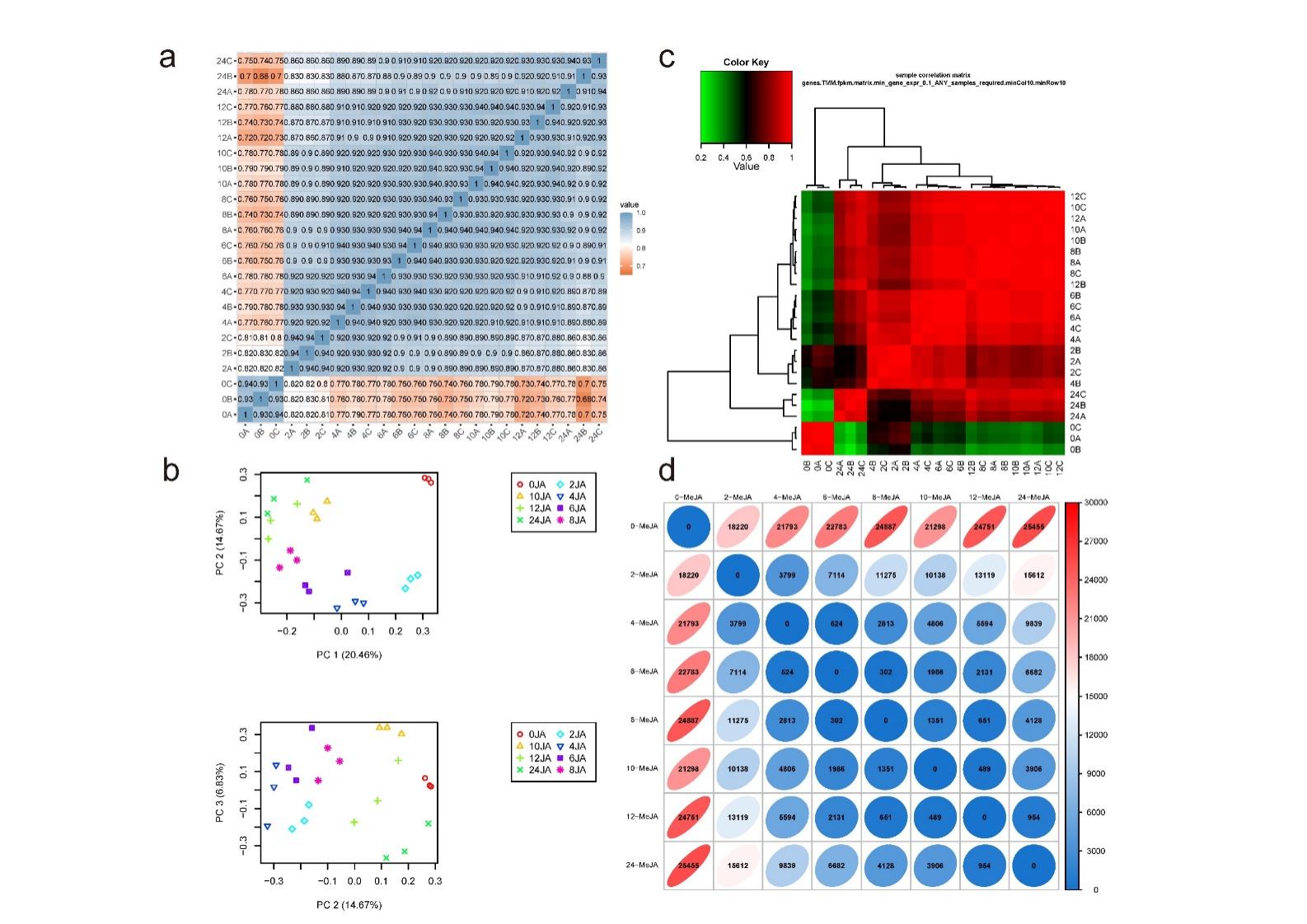


**Figure S2.** Quantification and differentially expressed genes (DEGs) of the samples. **a** Pearson correlation analysis of samples. **b** Principal component analysis of samples. **c** Cluster analysis of the samples. **d** DEGs of the *Tanacetum cinerariifolium* MeJA treatment sample.


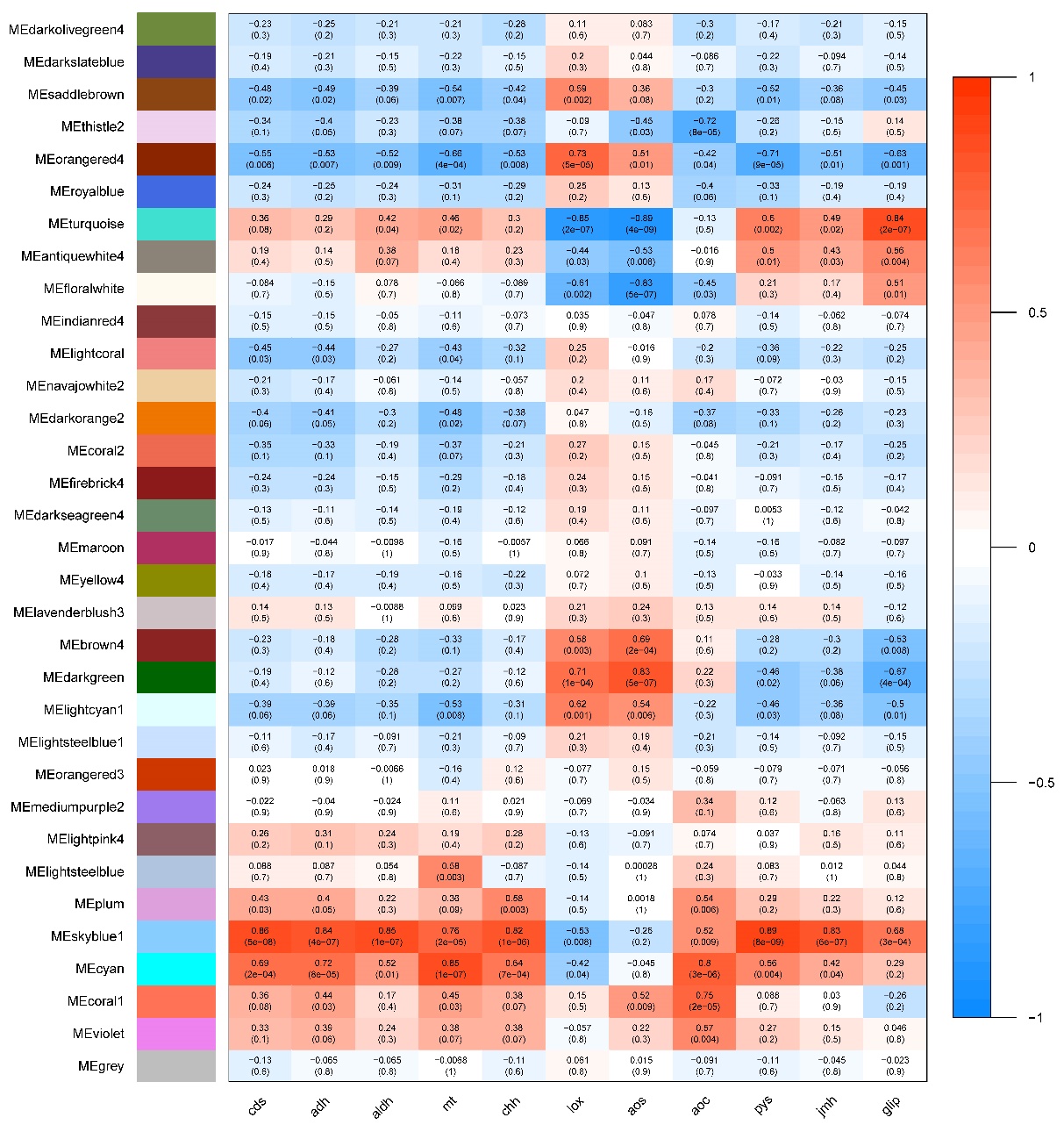


**Figure S3.** Coexpression modules identified by the WGCNA analysis.


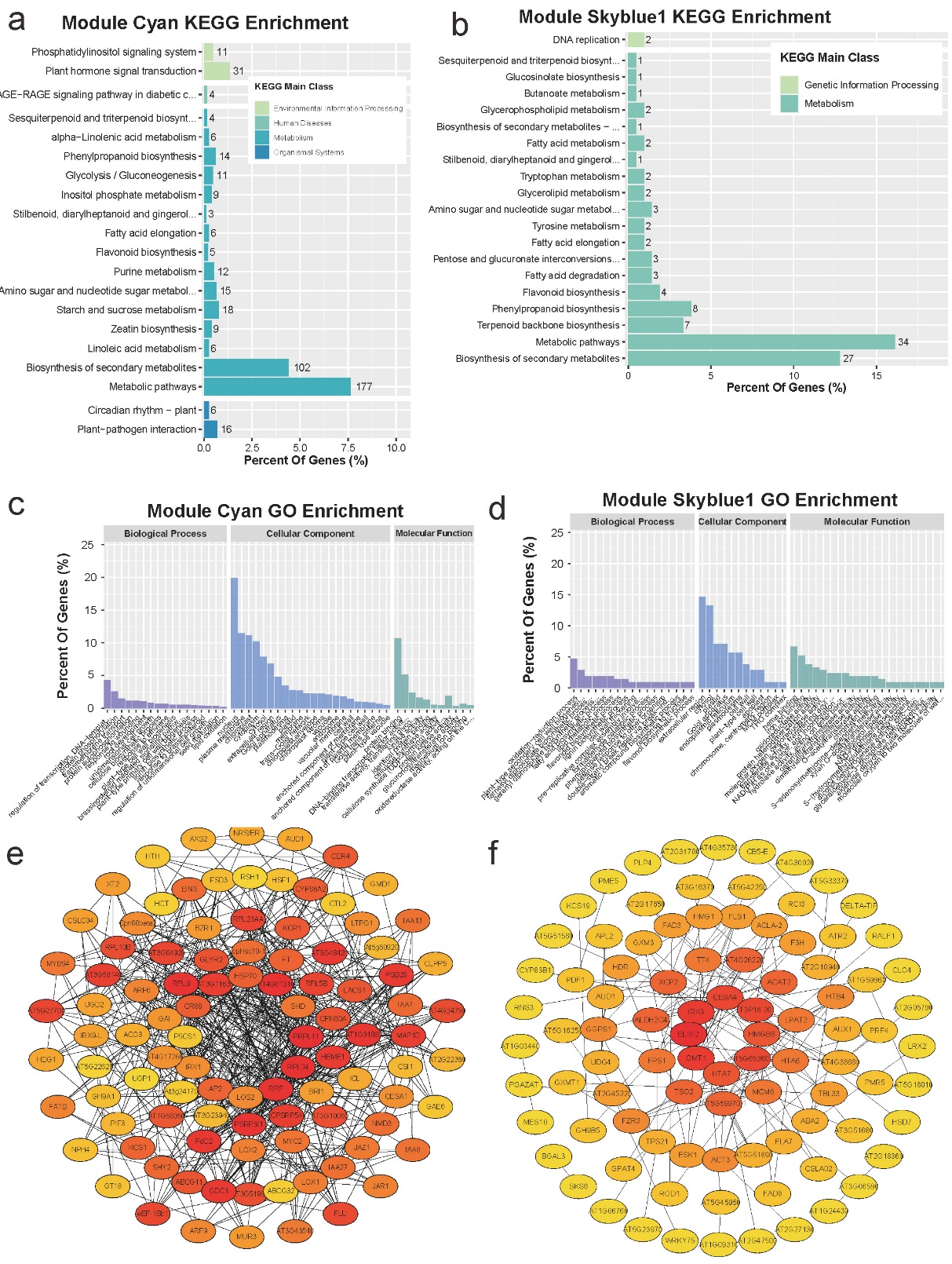


**Figure S4.** WGCNA function analysis and correlation network. **a** KEGG enrichment analysis of the Cyan module; **b** KEGG enrichment analysis of the Skyblue1 module; **c** GO enrichment analysis of the Cyan module; **d** GO enrichment analysis of the Skyblue1 module**; e** correlation network of the Cyan module; **f** correlation network of the Skyblue1 module.


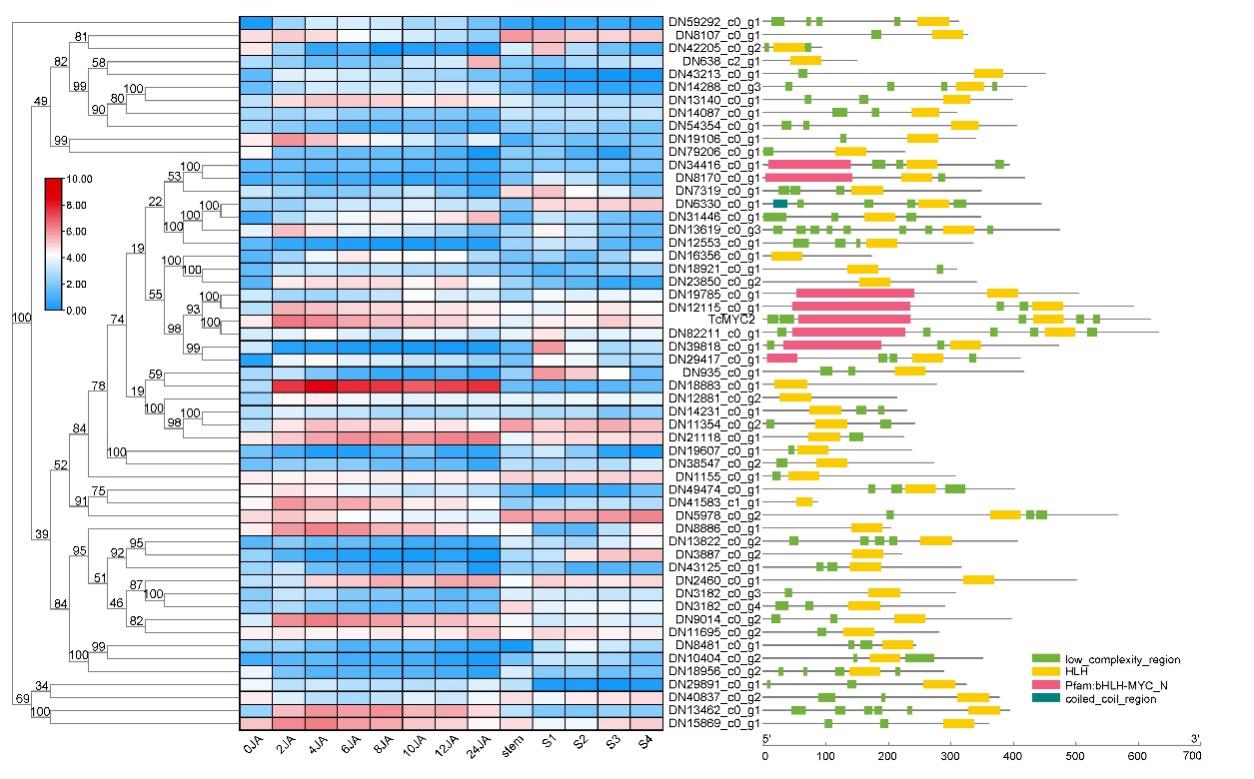


**Figure S5.** Phylogenetic tree, expression heatmap, and domains of the *Tanacetum cinerariifolium bHLH* family. All genes were detected by PlantTFDB, filtering out genes with average FPKM < 1 and lacking a bHLH domain. Sequences were aligned with maftt software. The maximum-likelihood phylogenetic tree was constructed using iqtree2 with 1000 bootstrap replicates. Numbers at nodes are bootstrap support values. Colors in the heatmap represent the value of log_2_(FPKM+1). The domains in the righthand figure were annotated with the SMART database.


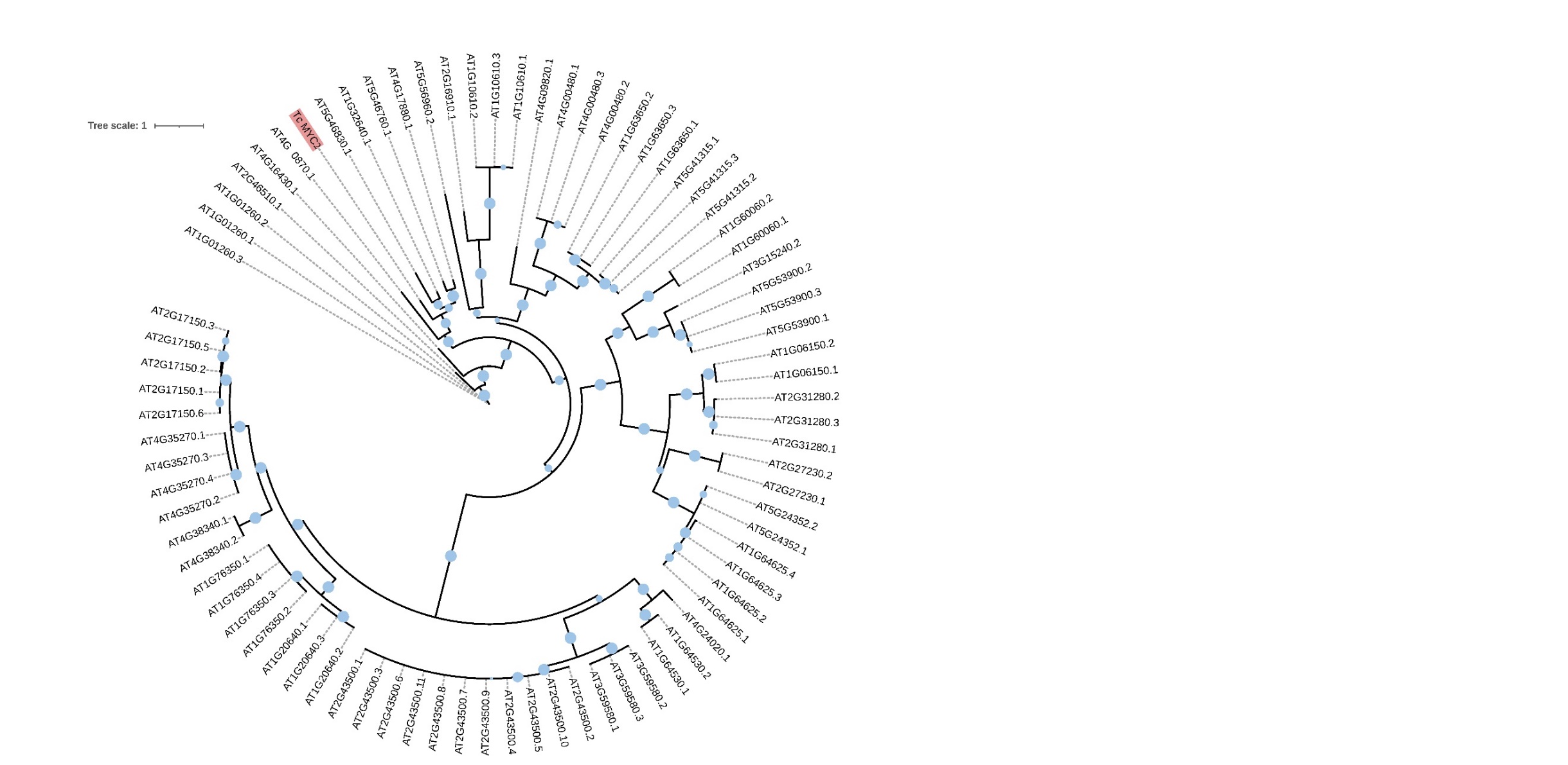


**Figure S6.** Phylogenetic tree of TcMYC2 and the *Arabidopsis thaliana* MYC family. The MYC proteins from *Arabidopsis thaliana* were scanned using HmmerSearch software with the Pfam domain database. The coding sequences were aligned with mafft software. The maximum likelihood phylogenetic tree was constructed using iqtree2 with 1000 bootstrap replicates. The circle size at a node indicates the level of bootstrap support. The scale bar is the number of amino acid substitutions per site.

| **Table S1. Primers used in this study** | | |
| --- | --- | --- |
| ID | Primer name | Primer sequences(5' to 3') |
| Realtime PCR | TcJMH_RT_F | ACTGCTTTCGAGATTTAGATTTGTTTTA |
| Realtime PCR | TcJMH_RT_R | CTTTTCTCATCACTTTCGGGTTCTTCA |
| Realtime PCR | TcLOX_RT_F | AATGCGCCGGTTACTCCCTGAC |
| Realtime PCR | TcLOX_RT_R | AAAACGCGTAAAAAGCTTCCACAATCT |
| Realtime PCR | TcAOC_RT_F | TTCAAGGCGCATACATCACAACT |
| Realtime PCR | TcAOC_RT_R | GTCACCGGGGTAACTAACAACTCA |
| Realtime PCR | TcCHS_RT_F | ACGTGCATCTTCTGGACCTCTTC |
| Realtime PCR | TcCHS_RT_R | TGAACAATCCGACGGTTAAGAGTC |
| Realtime PCR | TcADH2_RT_F | CTCGTATTCCTGGACATGAAGGTG |
| Realtime PCR | TcADH2_RT_R | GGCATCACTATGTCTCCTGGTTTC |
| Realtime PCR | TcALDH1_RT_F | CATTCCGCTACTTTGCTGGTGC |
| Realtime PCR | TcALDH1_RT_R | TCCAAGGAATGATGTGTCCAACTAC |
| Realtime PCR | TcGLIP_RT_F | GCCGGGAATGCGAGCAAAACAAC |
| Realtime PCR | TcGLIP_RT_R | CGCTCTCGCCTTCCTTAAAACCATA |
| Realtime PCR | TcCHH_RT_F | CCACGTGTATGTAGGGAGCAATGC |
| Realtime PCR | TcCHH_RT_R | CAGGATCAGTTGAACATGCGAAAG |
| Realtime PCR | TcMT_RT_F | GCTACGAGCTGTGGTAGAACCAATG |
| Realtime PCR | TcMT_RT_R | CTCTATAGCCACACGTTCTTCGAGC |
| Realtime PCR | TcPYS_RT_F | CTACAAGGTGCCCCCAAAGA |
| Realtime PCR | TcPYS_RT_R | TGATGACGGCTCCAAGGTAGA |
| Realtime PCR | TcGAPDH_RT_F | AAGGAGGAATCTGAAGGAAAGCTG |
| Realtime PCR | TcGAPDH_RT_R | GTTGTTGTTCAAAGCGATTCCAGC |
| Realtime PCR | TcMYC2_RT_F | TTCTGATACCACGGCTACTA |
| Realtime PCR | TcMYC2_RT_R | GCCAGACAATCCTTGATGAT |
| Linking to pHis2.1 vector | GLIP_Pro_pHis_F | gactcactatagggcgaattcAAACTAGAAGCAAAGATCATCGTACTTC |
| Linking to pHis2.1 vector | GLIP_Pro_pHis_R | attactagtggatccacgcgtAGCTTATATGTGCTCAGACAAGAGGT |
| Linking to pHis2.1 vector | AOC_Pro_pHis_F | gactcactatagggcgaattcTATTTAACTTGTATATATACATGGGTTGAAGC |
| Linking to pHis2.1 vector | AOC_Pro_pHis_R | attactagtggatccacgcgtAGTTGTTAAGATTTGTTTTAATGTTTAATGC |
| Linking to pHis2.1 vector | ALDH_Pro_pHis_F | gactcactatagggcgaattcCCCCTCTATAGAAAGATAATTTAATTCTCG |
| Linking to pHis2.1 vector | ALDH_Pro_pHis_R | attactagtggatccacgcgtTTTTCTCCTCTCTCTCTCTTTTTTTAATT |
| Linking to pHis2.1 vector | CHS_Pro_pHis_F | gactcactatagggcgaattcGCTATTATAAAATCCCGTGTCTATGC |
| Linking to pHis2.1 vector | CHS_Pro_pHis_R | attactagtggatccacgcgtCATTTACAACAGAATCTTAATGTGAGTGT |
| Linking to pGADT7 vector | MYC2_pGADT7_F | gtaccagattacgctcatatgATGACGATGAACATATGGAATTCAG |
| Linking to pGADT7 vector | MYC2_pGADT7_R | acgattcatctgcagctcgagCTACCCATGAGAAAGACAAGCATACC |
| Linking to pSuper1300GFP vector | MYC2_s1300g_F | gggcccggggtcgacatttaaatATGACGATGAACATATGGAATTCAG |
| Linking to pSuper1300GFP vector | MYC2_s1300g_R | gcccttgctcaccatggtaccCCCATGAGAAAGACAAGCATACC |
| Linking to pGreenⅡSK62 vector | MYC2_SK62_F | caggaattcgatatcaagcttATGACGATGAACATATGGAATTCAG |
| Linking to pGreenⅡSK62 vector | MYC2_SK62_R | gtcgacggtatcgataagcttCTACCCATGAGAAAGACAAGCATACC |
| Linking to pGreenⅡ0800 LUC vector | AOC_LUC_F | gtcgacggtatcgataagcttTATTTAACTTGTATATATACATGGGTTGAAGC |
| Linking to pGreenⅡ0800 LUC vector | AOC_LUC_R | caggaattcgatatcaagcttAGTTGTTAAGATTTGTTTTAATGTTTAATGC |
| Linking to pGreenⅡ0800 LUC vector | CHS_LUC_F | gtcgacggtatcgataagcttGCTATTATAAAATCCCGTGTCTATGC |
| Linking to pGreenⅡ0800 LUC vector | CHS_LUC_R | caggaattcgatatcaagcttTTACAACAGAATCTTAATGTGAGTGTATGT |
| Linking to pGreenⅡ0800 LUC vector | ALDH_LUC_F | gtcgacggtatcgataagcttCCCCTCTATAGAAAGATAATTTAATTCTTG |
| Linking to pGreenⅡ0800 LUC vector | ALDH_LUC_R | caggaattcgatatcaagcttTTTTCTCCTCTCTCTCTCTTTTTTTAATT |
| Linking to pGreenⅡ0800 LUC vector | GLIP_LUC_F | gtcgacggtatcgataagcttAAACTAGAAGCAAAGATCATCGTACTTC |
| Linking to pGreenⅡ0800 LUC vector | GLIP_LUC_R | caggaattcgatatcaagcttAGCTTATATGTGCTCAGACAAGAGGT |
| Linking to pET6HN-C vector | MYC2_pET6N_F | gatctctaagcttgcgaattcTATGACGATGAACATATGGAATTCAG |
| Linking to pET6HN-C vector | MYC2_pET6N_R | accaggcggccgccagaattcGCCCCATGAGAAAGACAAGCATAC |
| Probes used in EMSA | Probe_CHS_Pro_F | TTTGAAGGCAAGTGATGTAAAGTGCTAAGTGTTAAGTCAATGATTATAT |
| Probes used in EMSA | Probe_CHS_Pro_R | ATATAATCATTGACTTAACACTTAGCACTTTACATCACTTGCCTTCAAA |
| Probes used in EMSA | Probe_GLIP_Pro_F | CCTGACAGTTGCTATTTAGTGCTGTCTACTTGTTTAGTTGTGGAGCAAATGACT |
| Probes used in EMSA | Probe_GLIP_Pro_R | AGTCATTTGCTCCACAACTAAACAAGTAGACAGCACTAAATAGCAACTGTCAGG |
| Probes used in EMSA | Probe_AOC_Pro_F | CAACTAATCACACACCGCCACGTGTCCACCGTCCACCTACT |
| Probes used in EMSA | Probe_AOC_Pro_R | AGTAGGTGGACGGTGGACACGTGGCGGTGTGTGATTAGTTG |
| Probes used in EMSA | mProbe_CHS_Pro_F | TTTGAAGGTCCTGTATGTAAAGTGCTAAGTGTTAAGTCAATGATTATAT |
| Probes used in EMSA | mProbe_CHS_Pro_R | ATATAATCATTGACTTAACACTTAGCACTTTACATACAGGACCTTCAAA |
| Probes used in EMSA | mProbe_AOC_Pro_F | CAACTAATCACACACCGCTCTTCTTCCACCGTCCACCTACT |
| Probes used in EMSA | mProbe_AOC_Pro_R | AGTAGGTGGACGGTGGAAGAAGAGCGGTGTGTGATTAGTTG |
| Probes used in EMSA | mProbe_GLIP_Pro_F | CCTGATCTCGTCTATTTAGTGCTGTCTACTTGTTTAGTTGTGGAGTCCCGTACT |
| Probes used in EMSA | mProbe_GLIP_Pro_R | AGTACGGGACTCCACAACTAAACAAGTAGACAGCACTAAATAGACGAGATCAGG |
| Linking to pTRV2 vector | MYC2_VIGS_F | agaaggcctccatggggatccGACACCTAATCCAAATAACCCGA |
| Linking to pTRV2 vector | MYC2_VIGS_R | cgtgagctcggtaccggatccCCATGAGAAAGACAAGCATACCTAGG |
| Linking to pBI121 vector | MYC_pBI121_F | acgggggactctagaggatccATGACGATGAACATATGGAATTCAG |
| Linking to pBI121 vector | MYC_pBI121_R | cgatcggggaaattcgagctcCTACCCATGAGAAAGACAAGCATACC |
| Primers used in hairy roots | RolB_F | GCTCTTGCAGTGCTAGATTT |
| Primers used in hairy roots | RolB_R | GAAGGTGCAAGCTACCTCTC |
| Primers used in hairy roots | 35s-TcMYC2_F | GACGCACAATCCCACTATCC |
| Primers used in hairy roots | 35s-TcMYC2_R | GCCAGACAATCCTTGATGAT |
